# Identifying weak interdomain interactions that stabilize the supertertiary structure of the N-terminal tandem PDZ domains of PSD-95

**DOI:** 10.1038/s41467-018-06133-0

**Published:** 2018-09-13

**Authors:** Inna S. Yanez Orozco, Frank A. Mindlin, Junyan Ma, Bo Wang, Brie Levesque, Matheu Spencer, Soheila Rezaei Adariani, George Hamilton, Feng Ding, Mark E. Bowen, Hugo Sanabria

**Affiliations:** 1 Department of Physics and Astronomy, Clemson University, Clemson, SC USA; 2 Department of Physiology and Biophysics, Stony Brook University, Stony Brook, NY USA; 3 Department of Chemistry, Clemson University, Clemson, SC USA; 4 Center for Optical Materials Science and Engineering Technology, Clemson, SC USA

## Abstract

Previous studies of the N-terminal PDZ tandem from PSD-95 produced divergent models and failed to identify interdomain contacts stabilizing the structure. We used ensemble and single-molecule FRET along with replica-exchange molecular dynamics to fully characterize the energy landscape. Simulations and experiments identified two conformations: an open-like conformation with a small contact interface stabilized by salt bridges, and a closed-like conformation with a larger contact interface stabilized by surface-exposed hydrophobic residues. Both interfaces were confirmed experimentally. Proximity of interdomain contacts to the binding pockets may explain the observed coupling between conformation and binding. The low-energy barrier between conformations allows submillisecond dynamics, which were time-averaged in previous NMR and FRET studies. Moreover, the small contact interfaces were likely overridden by lattice contacts as crystal structures were rarely sampled in simulations. Our hybrid approach can identify transient interdomain interactions, which are abundant in multidomain proteins yet often obscured by dynamic averaging.

## Abstract (web summary)

Biologically relevant weak and transient interdomain interactions within proteins are difficult to analyze. Here, the authors combine multiscale molecular dynamics simulations and high-precision FRET experiments to characterize interactions between the tandem PDZ domains of PSD-95, revealing previously hidden conformational states.

## Introduction

Intramolecular interactions within the primary amino acid sequence drives polypeptides to fold. High-affinity interactions, such as those forming the hydrophobic core, produce a relatively static conformation. Weak intramolecular interactions permit a dynamic ensemble of alternate conformations, which is difficult to predict and challenging to identify experimentally. Prediction of tertiary protein folding is now quite accurate for small protein domains. However, many proteins contain independently folded subdomains that subsequently assemble into a supertertiary structure^1^. For the folding of multidomain proteins, the subdomains act as the primary sequence and their intramolecular interactions drive supertertiary folding. The same forces govern tertiary and supertertiary folding. However, the surface of folded subdomains is generally polar resulting in low affinity intramolecular interactions. Hence, the resulting supertertiary structure may sample different conformations with similar free energy over a broad range of timescales.

Structure determination of multidomain proteins remains a major challenge because of their dynamic and heterogeneous nature. Thus, many structural biology methods cannot describe supertertiary proteins. Moreover, the size of multidomain proteins, and the long dynamic timescales limit traditional all-atom molecular dynamics (MD) simulations. As such, few computational methods can predict supertertiary structure, even when all subdomains structures are known^2,3^. Förster Resonance Energy Transfer (FRET) is not bound by these limitations so it can probe the structures of dynamic biomolecules in vitro and in vivo^4–9^.

**Fig. 1:**
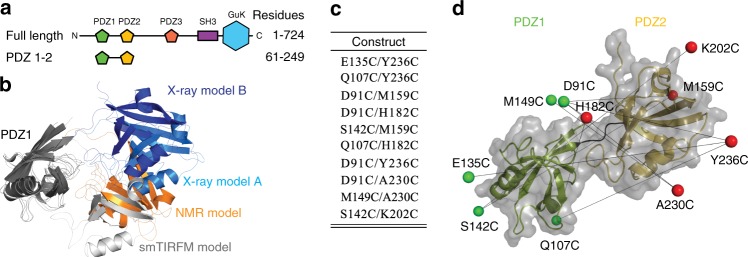
The PDZ1-2 tandem from PSD-95. **a** PSD-95 contains five protein-interaction domains connected by linkers of varying length. The N-terminal PDZ tandem contains the first two PDZ domains (residues 61-249). **b** Structural models of the PDZ tandem determined with different experimental methods. PDZ1 (gray) from each model was aligned to highlight conformational differences. PDZ2 is colored according the method used to resolve the structure. Models from the crystal structure (blue and cyan) [PDB ID: 3GSL]. Model from NMR based on residual dipolar coupling, which was kindly provided by M. Zhang (orange). Model from smFRET (white). **c** Cysteine mutations used for fluorescent labeling in the PDZ tandem of PSD-95 (Residues 60-249). The first residue is in PDZ1 while the second is in PDZ2. Each pair forms a single distance restraint that is measured independently. **d** Network of FRET restraints in the PDZ Tandem. Mean position of each dye (spheres) at the associated cysteine residue as determined by modeling. PDZ1 sites colored green. PDZ2 sites colored red. Black lines are shown between mean positions of dyes

Here, we present an integrative approach to supertertiary structure determination combining simulations and FRET experiments applied to the postsynaptic density protein of 95 kDa (PSD-95, Fig. 1a), which is a prototypical dynamic, multidomain protein^10–14^. PSD-95 contains five independently folded subdomains: three tandem PDZ domains, an SH3 domain and a Guanylate kinase-like domain, which are all involved in protein interactions at excitatory synapses^15,16^. The structures of *all* five subdomains are known^14,17,18^. Moreover, the PDZ domains of PSD-95 bind critical synaptic proteins such as ionotropic glutamate receptors, neuronal nitric oxide synthase^19^, the synaptic adhesion-protein neuroligin and synGAP, a GTPase linked to synaptic plasticity^12^. Previously, we probed the supertertiary structure of PSD-95 with single-molecule FRET^20^ revealing that the first two PDZ domains form a structurally independent supramodule^21^. There is particular interest in this PDZ1-PDZ2 tandem, which is the target of pharmaceutical compounds in clinical trials to treat ischemic stroke^22,23^.

PDZ domains independently fold into a conserved tertiary structure^16^. They appear within multidomain proteins, often in tandem, with up to 13 PDZ domains in a single protein^11,24^. The Protein Data Bank contains nearly 500 structures of PDZ domain but less than 30 are of tandem PDZ domains and none contain more than two domains. Thus, we lack information about the supertertiary PDZ interactions, which influence their function. PDZ domains typically interact with C-terminal peptides. As such, tandem PDZ domains are involved in multiprotein complex formation and often linked to signal transduction.

Studies of tandem PDZ domains revealed a range of interdomain affinities, which affect their supertertiary dynamics. For example, the Mint2 PDZ tandem was sufficiently dynamic that only the first domain of the tandem was seen as ordered within a protein crystal^25^. In contrast, intramolecular interactions within Mint1 lock the PDZ tandem into a single conformation that results in autoinhibition of peptide binding^26^. Thus, supertertiary interactions are an important regulatory mechanism for tandem PDZ domains.

NMR observed weak interdomain affinity within the PSD-95 PDZ tandem^27^ while FRET detected no interactions between the domains when unlinked^21^. Despite the weak interdomain interactions, NMR data suggested a “restrained conformation” in the unbound state, but identified no interdomain contacts to account for this restraint^27^ (Fig. 1b). In contrast, peptide binding unlocked the PDZ tandem resulting in a “dramatic change of protein dynamics”^28^. Thus, interdomain interactions appear incompatible with peptide binding suggesting the potential for autoinhibition of PSD-95. The PDZ tandem was also crystalized, which inherently suggests limited dynamics^29^. The crystal contained two different conformations (Fig. 1b). However, both lacked interdomain contacts that could explain the restricted dynamics. Finally, single molecule Total Internal Reflection FRET Microscopy (smTIRFM) experiments also observed limited dynamics but pointed to a model for the mean-occupied conformation that similarly lacked interdomain contacts (Fig. 1b)^21^. However, the time resolution (10 Hz) was insufficient to identify limiting conformational states.

Crystallography^29^, NMR^27^, and smTIRFM^21^ all suggest limited dynamics in the PDZ tandem, but the experimentally observed conformations show large divergence (Fig. 1b). Importantly, none of the models account for the restrictions on mobility suggesting that a limiting state with the PDZ domains in contact remains unknown. Each of these studies captured a different snapshot, but none provided a complete picture of the energy landscape.

To resolve the elusive limiting conformational states and detect supertertiary dynamics, we revisited FRET experiments on the PSD-95 PDZ tandem with orders of magnitude faster in time resolution. Additionally, we used Discrete Molecular Dynamics (DMD) simulations to map the energy landscape and identify interdomain interactions that stabilize the PDZ tandem. Experiments and simulations identified two limiting conformations for the PDZ tandem that involve different interdomain interactions: an open-like (OL) conformation characterized by a small contact interface area of 440 Å^2^ stabilized by interdomain salt bridges, and a closed-like (CL) conformation characterized by a larger contact interface with 701 Å^2^ of buried surface area, which is stabilized by surface-exposed hydrophobic residues. DMD suggests a relatively low-energy barrier that permits interconversion between these states, which fall within a relatively limited conformational space. Multiparameter fluorescence detection (MFD) confirmed the presence of submillisecond dynamics. Using disulfide mapping, we confirmed the location of the CL interdomain interface while salt-bridge mutations confirmed the OL interdomain interface. Thus, our hybrid approach, combining simulations and experiments, was able to resolve the two closely related conformations that confounded previous structural studies.

## Results

### A network of FRET restraints to probe supertertiary dynamics

We used a set of ten PDZ tandem variants with one unique cysteine in each PDZ domain (Fig. 1c), which were fluorescently labeled for FRET measurements. The labeling positions visually maximized the spatial distribution across the PDZ tandem within the constraints of surface accessibility and maintaining tertiary structural stability (Fig. 1d). The uncertainty in FRET-based structural models strongly depends on the number of restraints^30^. Assuming the domains to be rigid bodies, ten variants should provide sufficient restraints to determine the supertertiary structure of the PDZ tandem.

To minimize double labeling with the donor, which complicates data interpretation, PDZ tandem variants were sub-stoichiometrically labeled with donor. For a donor-only (DOnly) control sample, an aliquot of this reaction was quenched. The remaining sample was labeled with an excess of the acceptor (Alexa Fluor 647). Thus, the resulting samples are randomly labeled with a ~1:4 ratio of donor and acceptor fluorophores. However, the doubly labeled samples still contained a fraction of apparent DOnly protein, presumably due to the presence of an inactive acceptor^31^.

### Identification of limiting conformational states

**Fig. 2:**
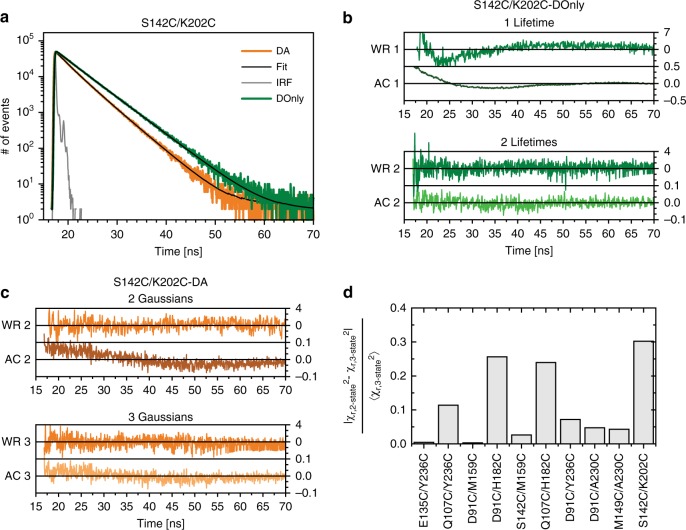
Ensemble time correlated single photon counting (eTCSPC) to identify limiting states. Representative data are shown for the variant S142C/K202C. The remaining variants are shown in Supplementary Fig. 1. **a** Time-resolved fluorescence decays for the Donor-only control (DOnly, green) and Donor-Acceptor FRET sample (DA, orange). Instrument response function is shown in gray. DA decays were fit using a global analysis of all ten variants (black lines). **b** Weighted residuals (WR, green) and autocorrelation (AC, lighter green) for fitting the DOnly decays with one (top panel) and two (bottom panel) fluorescence lifetimes. **c** Weighted residuals (WR, orange) and autocorrelation (AC, brown and cream) for the global fit using either two (top panel) or three (bottom panel) Gaussian-distributed states (Supplementary Table 1). **d** Improvement in χ2 when fitting with two or three Gaussian-distributed states. The relative change of χ2 is shown for each FRET variant (labels beneath panel)

By exciting the dyes with picosecond laser pulses, ensemble time correlated single photon counting (eTCSPC) can detect structural states that persist on the timescale of fluorescence emission (ns). Interdomain motions are much slower (µs-ms) so the PDZ tandem is essentially static during a round of fluorescence emission. The flexibly tethered dyes move at timescales slightly faster than the fluorescence emission, which uncouples motion of the dyes from the tandem^32^. We measured fluorescence lifetime decays for the 10 FRET variants and the 10 corresponding DOnly controls (Fig. 2a and Supplementary Fig. 1). To describe the donor de-excitation, we compared fitting the DOnly controls with a model containing one- or two-exponential decays (Supplementary Eq. 1). The weighted residuals and the autocorrelation of the residuals improved with two-exponential decays (Fig. 2b). We used the DOnly models to fit the corresponding DA sample under the assumption that all donor states are quenched equally by FRET. Each conformation has an associated rate of energy transfer (*k*_RET_) so the full decay is a superposition of the conformational states that are present (Supplementary Eqs. 3 & 4). Thus, eTCSPC results provide the number of conformations along with a model of the interdye distances for each conformation^5,33^.

To arrive at the correct number of conformations, we fit eTCSPC decays individually with an increasing number of states, and obtained the best results with three conformational states. The model also included a no FRET state to account for inactive acceptor molecules^31^. Thus, each DA sample yielded three interdye distances that arose from each of the conformational states. However, to use these FRET distances for modeling, it is necessary to assign each distance to its conformational state, which is typically done by population assignment. In this case, the states were distributed somewhat uniformly, which complicated the assignment. To ensure a consistent population distribution across all FRET samples and a proper assignment of states, we implemented a global fit for all ten FRET variants with a shared population distribution across the three states. The DA fluorescence decays were best described by three states based on improvements in the residuals (Fig. 2c and Supplementary Fig. 1). Moreover, the figure of merit (χ2) improved from an average of 1.26 to 1.15 (Supplementary Fig. 1K). For four variants, the improvement in χ2 was above 10% (Fig. 2d). The improvement in χ2 with three states was statistically significant based on the F-test (Supplementary Eq. 15)^34,35^ with a confidence interval greater than 99.9%.

With the global fit, we could simultaneously describe all 10 FRET decays with a unified, unbiased model. The most-populated state accounts for 43.5% of the population with 32.4% in the second-most-populated state and 24.1% in the least-populated state (Supplementary Table 1). Thus, the PDZ tandem samples three conformational states that give rise to different interdye distances.

The two main sources of uncertainty in the reported distances are: (i) the statistical uncertainty of fitting, and (ii) the uncertainty regarding the orientation of the dyes (*κ*^2^), which is an important parameter for converting FRET to distance. We estimated *κ*^2^ uncertainty from the anisotropy decays of the DOnly and DA samples including the directly excited acceptor and acceptor-sensitized emission (Supplementary Fig. 2). The *κ*^2^ distribution was calculated with a wobbling-in-cone model^36^ and compared to the standard assumption of 〈*κ*^2^〉 = 2/3 (Supplementary Fig. 3). We used these sources of error to determine the accuracy of our reported distances (Supplementary Eq. 16; Supplementary Table 1).

### Experimental confirmation of dynamic averaging

To detect dynamic transitions between the states identified by eTCSPC, we used MFD, which simultaneously records the average fluorescence lifetime, intensity and anisotropy of the photons emitted by each molecule. The conformational changes that occur while the molecule diffuses through the confocal excitation volume affect the fluorescence lifetime and intensity measurements differently. If a molecule samples multiple states, the FRET intensity indicator (*F*_D_/*F*_A_) and the average fluorescence lifetime (〈τ_D(A)_〉_f_) would differentially reflect changes to the mean and variance of the photon counts, respectively. This shifts the peak in the 2D histogram from the expected value for a static molecule in a single state^37,38^. This analysis is similar to mean-variance histograms in patch clamp electrophysiology^39^. Thus, plotting *F*_D_/*F*_A_ against 〈τ_D(A)_〉_f_ per single-molecule event is a useful tool to identify dynamics^37^.

**Fig. 3:**
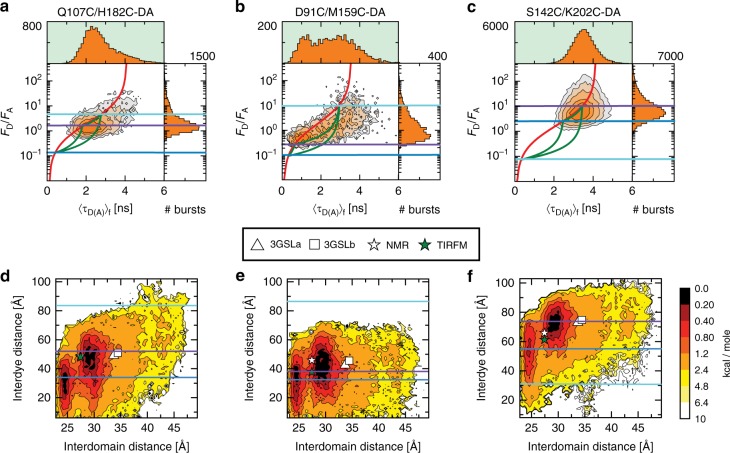
Multiparameter Fluorescence Detection of FRET and energy landscape of the PDZ1-2 tandem variants. Each panel plots two FRET indicators for each single-molecule event. The donor over acceptor fluorescence ratio (*F*_D_/*F*_A_) on the *y*-axis and the average fluorescence lifetime (〈τ_D(A)_〉_f_) on the *x*-axis. Shown are the 2D histograms (orange contours) for (**a**) Q107C/H182C-DA, (**b**) D91C/M159C-DA, and (**c**) S142C/K202C-DA. The 1D frequency histograms are shown along the axes for each FRET indicator. The static FRET lines (Supplementary Eq. 10, Supplementary Table 3) are shown in red. The dynamic FRET lines (Supplementary Eq. 11, Supplementary Table 4) are shown in green and connect the states identified with eTCSPC. The mean interdye distances for each of the eTCSPC states was converted to *F*_D_/*F*_A_ and shown as lines colored purple, blue, and cyan in decreasing order of population occupancy. **d**–**f** Histograms of the potential mean force (PMF) calculated from DMD simulations for the variants (**d**) Q107C/H182C-DA, (**e**) D91C/M159C-DA, and (**f**) S142C/K202C-DA. The remaining histograms are shown in Supplementary Fig. 6. Histograms show the interdomain distance between the center of mass of the two PDZ domains plotted against the interdye distance distribution, which was calculated as *p*(*R*_DA_)_PMF_ = exp(−*E*_PMF_/*k*_B_*T*) where *E*_PMF_ is the integrated free energy over interdomain distance as the function of the interdye distance, *R*_DA_; and *k*_B_ is Boltzmann constant. Contours are scaled in kcal/mole. Lines represent the mean interdye distances for each of the states identified by eTCSPC and are colored purple, blue, and cyan in decreasing order of population occupancy. Interdomain distances for previously reported models of the PDZ tandem are marked by symbols and denoted beneath the panels. Two basins are clearly identified, with residual states that are heterogeneously distributed with respect to the interdomain distance

For all 10 variants, we plotted *F*_D_/*F*_A_ against 〈τ_D(A)_〉_f_ as a 2D frequency histogram. The separate 1D frequency histograms for *F*_D_/*F*_A_ and 〈τ_D(A)_〉_f_ are aside and atop the main panel, respectively. Figure 3a–c shows three representative examples (A Q107/H182C-DA, B D91C/M159C-DA, and C S142C/K202-DA). For most variants, the frequency histograms appear as a single asymmetric population distribution (Supplementary Fig. 4).

We include guidelines to help interpret these frequency histograms. First, we calculated the expected *F*_D_/*F*_A_ for the three states identified by eTCSPC (purple, blue, and cyan lines in Fig. 3a–c). Next, we calculated the expected relationship between *F*_D_/*F*_A_ and 〈τ_D(A)_〉_f_  for molecules with no conformational dynamics (Fig. 3a–c red “static FRET” lines.). Finally, we calculated the expected relationship between *F*_D_/*F*_A_ and 〈τ_D(A)_〉_f_  for molecules undergoing transitions between states (Fig. 3a–c green “dynamic FRET” lines). Consequently, PDZ tandem molecules in a stable conformation will fall along the red static-FRET line while molecules undergoing conformational dynamics as they pass through the confocal excitation volume will fall off this line.

Given these guidelines, we observe that the Q107C/H182C variant is close to mid FRET (Fig. 3a) while D91C/M159C is centered at high FRET (low *F*_D_/*F*_A_, Fig. 3b), and S142C/K202C-DA shows low FRET (high *F*_D_/*F*_A_, Fig. 3c) consistent with previous measurements^21^. In these cases, the equilibrium lays closer to the most-populated state identified from eTCSPC (Fig. 3a–c, purple lines). Moreover, the population maxima for most variants fell within the area enclosed by the dynamic FRET lines indicating submillisecond dynamic averaging (Supplementary Fig. 4). It is important to note that at the time resolution of MFD, the limiting states from eTCSPC do not appear as discrete peaks due to averaging.

To confirm the presence of dynamic averaging, we varied the time binning of the data and used Photon Distribution Analysis (PDA)^38^ to model the *F*_D_/*F*_A_ distributions. For static molecules, the model function and χ2 would be unaffected by changing the time binning. In contrast, we observed that χ2 changed significantly when the time-binning window changed, which confirms dynamic averaging (Supplementary Fig. 5). Even our shortest time binning was unable to resolve the distribution into its underlying limiting states. This suggests that dynamics occur on timescales faster than the diffusion time, where time-binning analysis is optimal.

### DMD simulations of the PDZ tandem

To produce an unbiased representation of the conformational landscape, we performed MD simulations, which we compared with the experimental results. Given the size of the PDZ tandem and the relatively long timescale associated with domain motions, we used the atomistic DMD^40^. DMD is a rapid MD algorithm that has been benchmarked with ab initio protein folding^41^ and used to engineer de novo allosterically regulated proteins^42^ by capturing their coupled conformational dynamics.

As the starting conformation, we used the smFRET model^21^ and included the fluorescent dyes at all labeling sites to relate the simulations back to experiments. Importantly, the dyes were rendered non-interactive so as not to affect the simulation. To sufficiently sample conformational dynamics in the PDZ tandem, we performed replica-exchange DMD simulations with a cumulative total simulation time of ~1.6 μs. A previous benchmark study indicated that protein dynamics in DMD simulations with implicit solvent is two or three orders of magnitude faster than observed in experiments^43^. Hence, with efficient sampling enabled by DMD and the replica-exchange, we expected to observe dynamics beyond μs in experimental time. We applied the weighted histogram analysis method (WHAM)^44^ to analyze the conformational distribution from the replica-exchange trajectories, which give estimates of the potential mean force (PMF) at room temperature (300 K). We computed the PMF as a function of the interdomain distance between centers of mass of the two PDZ domains (*x*-axis, Fig. 3d–f and Supplementary Fig. 6). To facilitate a comparison between DMD simulations and experimental distances, we also computed the PMF as a function of the interdye distance for each FRET variant (*y*-axis, Fig. 3d–f and Supplementary Fig. 6). These two representations of the PMF are plotted against each other as a contour histogram in units of kcal/mole.

In this representation, we observed two distinct minima in the PMF energy landscape corresponding to two distinct conformational states along with a broad sampling of alternate conformations with larger interdomain distances. By projecting the free energy surface onto various reaction coordinates, we estimated the apparent energy barrier to be only a few kcal/mole (Fig. 3d–f and Supplementary Fig. 7 & 8), which is consistent with the dynamic averaging observed in MFD (Supplementary Fig. 4). Although the two conformations are reasonably separated in terms of interdomain distance, the associated interdye distributions are substantially overlapped for many variants (Supplementary Fig. 6). Both the simulated interdye distances (*p*(*R*_DA_)_PMF_) and, for most variants, the experimentally measured distances displayed a relatively unimodal distance distribution that did not resolve the two conformations into separate peaks (Supplementary Fig. 9). Thus, several variants were not sensitive to the conformational differences.

### Interdomain contacts that stabilize the PDZ tandem

**Fig. 4:**
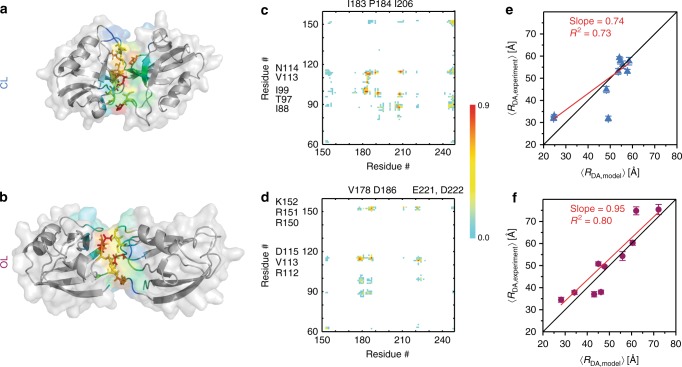
Representative structures, contact maps and correlation between experimental and DMD simulated interdye distances. **a** Cartoon representation for the closed-like (CL) state, which has the shorter interdomain distance and a larger contact interface. PDZ domains are shown in cartoon representation with a transparent surface. Interfacial residues are colored according to their contact frequency (cyan to red denoting low to high frequency). **b** Representative structure for the open-like (OL) state with increased interdomain separation and smaller contact interface. Rendered and colored as in panel **a**. **c** The interdomain contact frequency map per residue for the conformational ensembles of the CL state. Contact frequency uses same color palette as in panels A and B; no contact white, low contact probability cyan and high contact red. **d** The interdomain contact frequency map for the OL state. The color palette is the same as in (**c**). Residues with the highest interdomain contact frequencies are highlighted along the axis for each PDZ domain. **e**, **f** Measured interdye distances from eTCSPC (*y*-axis) plotted against the simulated interdye distances for the representative models shown in (**a**) and (**b**) (*x*-axis). **e** Distances for the CL state plotted against those for the second-most populated state in eTCSPC. **f** Distances for the OL state plotted against those for the most populated state in eTCSPC. Data was fit to a line to extract the slope and correlation. Error bars in (**e**) and (**f**) correspond to the uncertainty on the measured distances shown in Supplementary Table 1 using an error propagation rule (Supplementary Eq. 16)

The published structural models were unable to identify the interdomain contacts that stabilize the PDZ tandem. For the two identified minima, we selected a representative structure with the smallest average root mean squared displacement (RMSD) to the entire ensemble (Fig. 4a, b, respectively). The RMSD between the two representative models is 10.4 Å, which supports these being distinct conformations. The CL state model showed a buried surface area of 701 Å^2^. In contrast, the OL state model showed a smaller contact interface of 440 Å^2^. It should be noted that these representative structures fall within a rather shallow basin in the energy landscape. As such, these minimal surfaces do not represent the entire contact area for each conformational ensemble.

For each representative structure from the two basins identified in the energy landscape (Fig. 3d–f), we computed the frequency map of pair-wise residue contacts between the PDZ domains and plotted the location of persistent contacts (Fig. 4c–d). We found that the closed-like (CL) state is primarily stabilized by interactions between surface-exposed hydrophobic residues (highlighted next to the axes. Figure 4c). In contrast, the open-like (OL) state involved primarily electrostatic interactions between oppositely charged residues in PDZ1 and PDZ2 (Fig. 4d). An overlay of the two models highlights the rotation of PDZ2 with respect to PDZ1 that accompanies the transition between states (Supplementary Fig 10).

Having identified the interdomain contacts stabilizing each state, we computed the fraction of native contacts for each state (*Q*_CL_ and *Q*_OL_) and used these *Q*-values^45,46^ to represent the energy landscape at the room temperature. By tracking the formation of these interfaces, we observed multiple interconversions between states during our simulations (Supplementary Fig. 7). Moreover, *Q*-values showed that the two states occupy distinct basins in the energy landscape (Supplementary Fig. 7). Finally, the states are mutually exclusive as interdomain contacts in either state must break to transition to the other state (Supplementary Fig. 8).

### Comparison of simulations to the experimental distances

To compare DMD and eTCSPC, we added guidelines for distances of the three states from eTCSPC to the PMF plots from DMD (Fig. 3d–f and Supplementary Fig. 9). This shows that the most-populated and second-most populated states from eTCSPC generally correspond to the OL and CL states from DMD, respectively. The least-populated eTCSPC state, which has no direct corollary in DMD simulations, sampled a heterogeneous ensemble of states outside the predominant minima (Supplementary Fig. 8). Although DMD does not identify a discrete third state, we note that fitting the eTCSPC to a two-state model failed to provide positive correlations with the interdye distances from DMD simulations (Supplementary Fig. 10A). We conclude that the least-populated state (~24% of molecules) could encompass the wide range of outlying DMD conformations (~20% of the simulation time). The presence of outlying DMD states is readily apparent from the *Q*-value distributions, which reveals a population with Q-values corresponding to neither state (Supplementary Fig. 8B). This DMD “population” displayed a highly variable interdomain distance with no distinct minimum on the PMF histograms; hence, we are unable to reliably select a representative example for comparison with experiments.

To compare the experiments and simulations, we correlated the interdye distances from the DMD models to those from eTCSPC. The CL state showed a positive correlation indicating good agreement with an *R*^2^ of 0.73 and a slope of 0.74 (Fig. 4e). The OL state showed an *R*^2^ of 0.80 and slope of 0.95 representing even better agreement between experiment and simulation (Fig. 4f). The distribution of distances from DMD and MFD did show differences (Supplementary Fig. 9). This is not unexpected as variance in DMD arises from conformational sampling, while variance in MFD depends on signal to noise and the rate of conformational exchange. However, the mean interdye distances from MFD also correlated well with mean interdye distances from DMD with *R*^2^ = 0.80 (Supplementary Fig. 9K).

### Disulfide mapping to probe the predicted contact interface

**Fig. 5:**
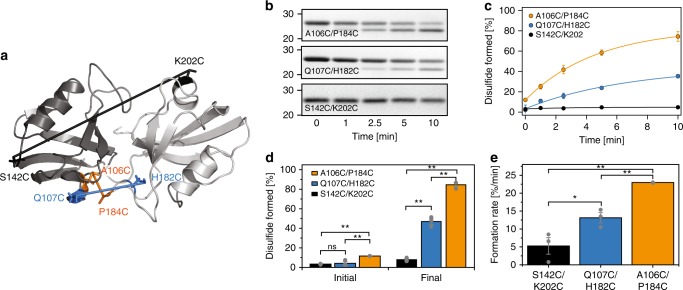
Disulfide mapping of the interdomain contact interface in the PDZ tandem. **a** Location of the engineered cysteine pairs used to test the contact interface. Cartoon representation of the CL state of the PDZ tandem. PDZ1, gray. PDZ2, white. Cysteine side chains are shown as lines with the pairs connected by their sulfur atoms. A106C/P184C, orange. Q107C/H182C, blue. S142C/K202C, black. **b** Representative SDS-PAGE showing individual time points in the disulfide bond formation reaction used for the analysis. Disulfide formation was initiated by shifting the samples to oxidizing conditions. The location of the introduced cysteine residues for each mutant is shown within each panel. Duration of the reaction for each time point is indicated below. The full gels are shown in Supplementary Fig. 11. **c** Kinetic analysis of the disulfide formation reactions. A106C/P184C, orange. Q107C/H182C, blue. S142C/K202C, black. Gel band intensities were measured in triplicate to obtain the percentage of disulfide for each time point. Each time course was fit with an exponential function to obtain the initial and final percentage of disulfide bonding along with the rate of disulfide formation. **d** Extent of disulfide formation for each mutant taken from the fits to panel B. The initial percentage (left) showed the presence of disulfide before the oxidation reaction. The final percentage showed the amount of disulfide formed after the oxidation reaction has gone to completion. ** indicates p > 0.01; ns stands for no significance in an unpaired student *t*-test. **e** Disulfide formation rate for each mutant as taken from the fits to panel b. ** indicates *p* > 0.002. * indicates *p* > 0.05 in an unpaired student *t*-test. For all graphs, error bars indicate SEM for three replicate measurements. Individual values are shown as dots

To validate our model for the CL state, we monitored the rate of disulfide formation between engineered cysteine residues at different positions within PDZ1 and PDZ2. Disulfide bond formation is distance dependent so the rate is determined by structural proximity^47^. Therefore, we introduced a disulfide pair at the predicted contact interface, A106C/P184C (Fig. 5a, orange). These residues are ~5 Å apart and should have the highest rate of disulfide formation. The proline is in a loop region so the mutation was well tolerated. As a positive control, we selected a FRET variant with the high FRET efficiency, Q107C/H182C (Fig. 5a, blue). These residues are ~15 Å apart so these residues should have reduced disulfide formation. As a negative control, we chose a FRET variant with low FRET efficiency, S142C/K202C (Fig. 5a, red). These residues are over 40 Å away and should form disulfides poorly.

We initiated disulfide formation with copper (II) phenanthroline followed by non-reducing SDS-PAGE, which revealed increased mobility upon disulfide formation (Fig. 5b). Additionally, a small amount of higher order disulfide formation occurred (Supplementary Fig. 11). For each variant, we measured the disulfide formation in triplicate (Fig. 5c). Each reaction was well fit to a single exponential function to obtain the initial and final extent of disulfide formation along with the reaction rate for each variant. Analysis of the extent of disulfide formation revealed that less than 5% of the control samples had formed disulfides before initiating the reaction, while the A106C/P184C mutant showed 11.6 ± 0.08% disulfide formation before the reaction had started (Fig. 5d). The negative control showed no significant disulfide formation during the reaction (initial 3.37 ± 0.32% and final 3.69 ± 5.06%) (Fig. 5d). The positive control showed 46.94 ± 2.83% disulfide formation. However, the mutant designed to be at the CL interface (A106C/P184C), showed the highest extent of disulfide formation (84.58 ± 2.01%). The differences in the extent of disulfide formation were mirrored by differences in reaction rates (Fig. 5e). Although the negative control showed minimal disulfide bond formation, we estimated the reaction rate to be 5.27 ± 2.31%/min. The positive control formed disulfides 2.5 fold faster (13.13 ± 1.4%/min). Thus, the disulfide formation rates correlate with differences in FRET efficiency. In contrast, the A106C-P184C mutant formed disulfides 4.4 fold faster (22.97 ± 0.04 %/min) and 1.7 fold faster than the positive control. Thus, the extent and rate of disulfide formation agree with predictions of proximity based on our CL state model. This confirms the predicted interdomain contact interface.

To probe the OL state, we engineered a salt bridge by introducing the mutations: V113K, N114K, V223D, and M224E into the variant Q107C/H182C (Supplementary Fig. 12). V113 and N114 are within the OL interface. Replacing these uncharged residues with lysines creates an electropositive patch on PDZ1. In contrast, replacing V223 and M224 with aspartate and glutamate creates an electronegative patch on PDZ2. V223D and M224E are not within the OL interface, but are close enough that the electrostatic interactions should disfavor the CL state. By measuring smFRET in MFD (Supplementary Fig. 12B-C), we observed a shift towards higher *F*_D_/*F*_A_ (i.e., lower FRET) indicating a larger interdye distance in the “salt-bridge” variant compared to the control. The domain rotation needed to bring complementary charges together would move these labeling sites farther apart. Thus, the observed FRET shift is consistent with the expected motion. Thus, by adding additional salt bridges to the ionic interactions in the OL state, we observe a clear redistribution of FRET.

In summary, by using a hybrid approach combining eTCSPC, DMD, and MFD, we provide a self-consistent view of the conformation and dynamics of the PDZ tandem, which we independently verified by experimentally probing the predicted interdomain contacts.

## Discussion

The PDZ1-2 tandem from PSD-95 has been extensively studied but none of the reported structures identified any interdomain contacts that explain the limited dynamics observed in these reports (Fig. 1). Here, we used replica-exchange DMD simulations, totaling 1.6 μs, to identify two predominant low-energy basins in the conformational landscape, which correspond to two limiting state structures that involve different interdomain contacts (Fig. 4). Our eTCSPC analysis identified three states (Fig. 2) with the two predominant states corresponding to those from DMD with a correlation coefficient above 73% (Fig. 4). MFD analysis confirmed the presence of dynamic averaging at timescales faster than milliseconds (Fig. 3a–c). Moreover, time window analysis showed significant changes in the figure of merit at different time binning (Supplementary Fig. 5). This is a clear signature of dynamic averaging at timescales faster than milliseconds. Therefore, MD simulations and experiments agree that the PDZ tandem is dynamic and samples two low-energy conformations that are similar but not identical.

The coexistence of two similar conformational states revealed the difficulty in designing a network of FRET distance restraints. Including the dyes in DMD simulations revealed that several restraints showed similar interdye distances for the two conformations. This lack of conformational sensitivity for some variants explains why treating measurements separately was less successful than a global fit at consistent assignment of state distributions. The two most populated states, which agree with DMD, account for 75.9% of the FRET observations. The remaining 24.1% show significant heterogeneity among variants and, in some cases, correspond to distances beyond those sampled by DMD. Despite these limitations, global treatment of the ten fluorescence decays provided a unified description of the two predominant conformational states.

Moreover, we validated both limiting states by (i) engineering a disulfide bridge in the interface of the CL state (Fig. 5) and (ii) enhancing the salt-bridge interactions that stabilize the OL state. Our measurements of these variants were in agreement with the expectations based on DMD (Fig. 5 and Supplementary 11). Thus, we independently corroborated the proposed structural models, which sets a new standard for accuracy in FRET-based protein modeling.

To compare previous structural models of the PDZ1-2 tandem with DMD, we calculated the interdomain distances for the two crystallographic models^29^, the representative NMR model^27^ and the smTIRFM model^21^. The interdye distances were estimated from the accessible volume for dyes in each structure. We mapped these interdomain and interdye distances on to the PMF histograms from DMD (Fig. 3d–f). For the crystallographic models, the interdomain distances (33.7 Å and 34.5 Å for 3GSLa and 3GSLb, respectively) are larger than the interdomain separation observed in DMD simulations (Fig. 3a–c). Thus, the crystal structures were infrequently sampled in simulations. The extensive crystal lattice interactions may have overriden the stabilization provided by the weaker intramolecular interface. For the NMR model, the interdomain distance falls between the two states and closer to the OL state, which was the most populated conformation in eTCSPC. The same is also true for the smTIRFM model, which was between the two states from DMD and similarly closer to the OL state. This is understandable given that the measurement timescale was slower than the submillisecond dynamic averaging observed in MFD. Thus, the dynamic exchange between two closely related conformations complicated the derivation of structural models by NMR and smTIRFM.

NMR^48^ and SAXS^23^ experiments showed that peptide binding to the PDZ tandem increased both interdomain flexibility and interdomain separation such that the defined orientation between PDZ domains was lost. Thus, peptide binding may be incompatible with the CL conformations we observed. Neither of the peptide binding sites in the PDZ tandem are sterically occluded in either state. However, the binding affinity of PDZ domains is known to be regulated through allosteric interactions^49^. Such allosteric networks have been identified operating in PDZ2 from PSD-95^50^. Moreover, ligand binding to PDZ domains is coupled to widespread changes in sidechain dynamics such that interactions distal from the binding pocket can affect affinity^51^. Closer examination of the two limiting states reveals that the contact interfaces involve residues that surround the ligand binding sites. PDZ domains bind ligands in the β2-α2 groove near the long α2-helix. The CL state interface involves residues in the β2-β3 loops of both domains, which are near the exit of the ligand-binding pocket. In the OL state, the β2-β3 loop and the β5 sheet, which abuts α2, interact with the interdomain linker and with β4 on the backside of PDZ1. Such interactions between elements around the binding sites could easily modulate affinity resulting in autoinhibition. Validation of autoinhibition in either state remains for future studies.

We combined ensemble and single-molecule FRET experiments with high temporal resolution and DMD simulations to describe the supertertiary structure and dynamics of the PDZ1-2 tandem from PSD-95. DMD simulations observed a heterogeneous free energy landscape with two clear minima. These two conformations were confirmed by eTCSPC while the temporal resolution of MFD showed that the PDZ1-2 tandem is dynamically exchanging. The upper limit of the dynamic timescale is set by time binning in MFD experiments, with a mean diffusion time of ~1 ms. Such fast interconversion is consistent with a low free energy barrier separating the two states as suggested by simulations. This suggests weak interdomain interactions, which is consistent with the relatively small contact areas observed for each state. While neither state was very stable, it is likely that the presence of two nearby contact states minimizes the time spent in unrestrained conformations associated with peptide binding. Our results explain the highly divergent models from previous structural studies.

Finally, our approach is uniquely positioned to study weak and transient interdomain interactions. Although often hidden from biophysical characterization, they are highly abundant in multidomain proteins such as poly-PDZ proteins^14^, ion channels^52^, ATPases^53^, enzymes^54^, and intrinsically disordered proteins with dynamic supertertiary structures^1^. When identified, these transient and short-lived conformational states could serve as potential targets for drug development and may reveal novel mechanisms of action^55–57^.

## Methods

### Protein expression and purification

The PDZ1-2 tandem from *Rattus norvegicus* PSD-95 (residues 61-249) was expressed from pPROEX HTB in the BL21 *E. coli* strain induced with 0.5 mM IPTG for 2 h at 30 °C. Proteins were purified by a Ni-affinity and eluted with 250 mM imidazole. The 6 His tag was removed by TEV protease cleavage during dialysis into 20 mM tris pH7.4 100 mM NaCl 1 mM DTT 1 mM EDTA followed by anion exchange using HiTrap Q columns (GE Healthcare). Finally, proteins were purified by size exclusion chromatography on Superdex 75 (GE Healthcare)^21^. Proteins were first labeled with a 1:2 ratio of Alexa 488 C_5_ maleimide for 1 h at 4 °C followed by addition of a 5:1 molar ratio of Alexa Fluor 647 C_2_ maleimide, which was reacted overnight at 4 °C. Unconjugated dye was removed by desalting with Sephadex G50 (GE Healthcare) followed by dialysis.

### Ensemble time correlated single photon counting

Ensemble time-correlated single-photon-counting (eTCSPC) measurements were performed using a Fluorolog3 spectrofluorometer in T format with a PDX detector and Horiba Yvon photon system (Horiba, USA). The excitation sources were a pulsed 485L and a 635L NanoLED diode lasers (Horiba Yvon, USA) operating at 1 MHz, for donor and acceptor, respectively. The emission monochromator was set to the maximum emission wavelength of the fluorophore being measured, while the slit was set at a maximum of 10 nm in order to get an intensity count rate lower than 2% of the repetition rate. Fluorescence decay analysis is done using ChiSurf written in Python following the algorithm described in Supplementary Methods.

### Multiparameter fluorescence detection

Freely diffusing molecules in pM concentration were excited as they passed through the focal volume of a 60 × , 1.2 NA collar (0.17) corrected Olympus objective. Pulsed interleaved excitation (PIE)^58^ with diode lasers at 485 nm and 640 nm (PicoQuant, Germany) was operated at 40 MHz with 25 ns interleaved time. The power at the objective was 120 µW at 485 nm and 39 µW at 640 nm. Emitted photons were collected through the same objective and spatially filtered through a 70 µm pinhole to limit the effective confocal detection volume.

Emission was separated into parallel and perpendicular polarization components at two different spectral windows using band pass filters ET525/50 and ET720/150 (Chroma Technology Co.) for donor and acceptor, respectively. Four photon-detectors are used—two for donor (PMA Hybrid model 40 PicoQuant, Germany) and two for acceptor (PMA Hybrid model 50, PicoQuant, Germany). To ensure temporal data registration of the four synchronized input channels, we used a HydraHarp 400 TCSPC module (PicoQuant, Germany) in Time-Tagged Time-Resolved mode.

Labeled samples were diluted to pM concentration in PBS buffer (50 mM sodium phosphate, pH 7.5, 150 mM NaCl, 40 μM TROLOX), which had been charcoal filtered to remove residual impurities. At pM concentrations, we observe ~1 molecule per second in the focal volume. Samples were imaged in NUNC chambers (Lab-Tek, Thermo Scientific, Germany) that were pre-coated with a solution of 0.01% Tween 20 (Thermo Scientific) in water for 30 min to minimize surface adsorption. We obtained the instrument response function (IRF) by measuring water while protein-free buffer was used for background subtraction. Calibration experiments and details on data collection were recently reported^59,60^.

### DMD simulations

A detailed description of the DMD algorithm can be found elsewhere^40,43^. Briefly, proteins were represented by the united-atom model containing all heavy atoms and polar hydrogens with an implicit solvent model. The step-wise potential functions were obtained by mimicking the continuous interatomic interaction potentials in the molecular mechanics-based Medusa force field^61^. The Medusa force field has been shown to be effective in accurate prediction of protein stability changes upon mutations for a large set of experimental data^62^. The bonded interactions include covalent bonds, bond angles, and dihedrals. The interatomic interactions include van der Waals, solvation, hydrogen bond, and electrostatics. The solvation energy is modeled by the Lazaridis-Karplus implicit solvent model^63^. An implicit solvent approach is appropriate for studying long timescale dynamics of biomolecules due to the separation from the fast water dynamics. Screened electrostatic interactions are computed by the Debye-Hückel approximation. A Debye length of 1 nm is used by assuming a water dielectric constant of 80 and a monovalent electrolyte concentration of 0.1 M. The Anderson’s thermostat is used to maintain constant temperature and a periodic boundary condition is applied. Because they follow the same physical laws, the dynamics observed in DMD are equivalent to continuous potential MD at timescales longer than picoseconds with differences mainly at short timescales within the sub-picosecond range (i.e., the average time step between two consecutive interatomic collisions where a potential energy step is encountered).

DMD simulations included the fluorescent dyes, which were coupled to the corresponding cysteine residues as in experiments. The atoms in the linker were rendered non-interacting by setting a very small hardcore distance with all other atoms (0.001 Å). The atoms within the dye moiety have a hard-sphere interaction with other atoms of the dye through a hardcore distance of 3 Å, while the dyes themselves are effectively non-interactive through a small hardcore distance of 0.001 Å

We performed replica-exchange DMD simulations with eight replicas at different temperatures: 275, 287, 300, 315, 330, 345, 360 and 375 K. The exchange between replicas with neighboring temperatures was carried out every 50 ps. Each replica lasted 200 ns with an accumulative simulation time of 1.6 μs. The PMF was calculated by the WHAM using the last 175 ns from each of the eight simulations. The DMD program is available online (www.moleculesinaction.com).

### Disulfide mapping in the PDZ1-2 tandem of PSD-95

Proteins were purified under reducing conditions. Immediately before the reaction, proteins were fully reduced by incubation in fresh 5 mM DTT for 1 h at 4 °C followed by desalting into non-reducing conditions (20 mM tris pH 7.4, 150 mM NaCl, 1 mM EDTA). Disulfide oxidation reactions were performed using a protein concentration of 2 μM at 25 °C. Disulfide formation was initiated by the addition of 0.5 mM CuSO_4_ and 1.75 mM 1, 10-phenanthroline. Time points were quenched by adding 40 mM N-ethylmaleimide to alkylate unbonded cysteines and 10 mM EDTA followed immediately by boiling at 95 °C for 5 min in non-reducing Laemmli sample buffer^47^. Samples were run on 15% SDS-PAGE. All experiments were carried out in triplicate. Intensities for both the native and shifted bands were measured in ImageJ. Percentages of disulfide formation were calculated for each time point and corrected for the presence of higher order oligomers. Each reaction was well fit to a single exponential function to obtain the initial and final extent of disulfide formation along with the reaction rate for each mutant. Replicates were analyzed separately to obtain the average and standard error of the mean (SEM) as well as to estimate the error in the fitted parameters.

### Code availability

MFD is made available at http://www.mpc.hhu.de/en/software. ChiSurf used for fluorescence decay analysis is available at http://www.fret.at/tutorial/chisurf/. DMD simulation engine is available at http://www.moleculesinaction.com.

## Electronic supplementary material

Supplementary Information

## Data Availability

The authors declare that all data supporting the findings of this study are available within the paper and its supplementary information file, and available from the corresponding authors upon reasonable request.
