# Supplementary material for: Identifying weak interdomain interactions that stabilize the supertertiary structure of the N-terminal tandem PDZ domains of PSD-95
Source: Nat Commun. 2018 Sep 13;9:3724. doi: 10.1038/s41467-018-06133-0 (PMC6137104; doi:10.1038/s41467-018-06133-0)
Supplement: Supplementary file 1 — Supplementary Information [file 41467_2018_6133_MOESM1_ESM.pdf]

Nature Communications: Supplement

Identifying Weak Interdomain Interactions that Stabilize the Supertertiary Structure of the N-Terminal Tandem PDZ Domains of PSD-95

Authors: Inna S. Yanez Orozco[1§], Frank Mindlin[2§], Junyan Ma[3,4], Bo Wang[1], Brie Levesque[2], Matheu Spencer[1], Soheila Rezaei Adariani[1], George Hamilton[1], Feng Ding[1], Mark E. Bowen[2], Hugo Sanabria[1,4*]
§: contributed equally, *: corresponding authors

	Title	Page
**Supplementary Figure 1**	Time Resolved Fluorescence of donor only (DOnly) and donor-acceptor (DA) labeled PDZ1-2 tandems.	**3**
**Supplementary Figure 2**	Time Resolved Fluorescence Anisotropy of donor only (DOnly) and donor-acceptor (DA) labeled PDZ1-2 tandems.	**4**
**Supplementary Figure 3**	κ^2 distribution using wobble in a cone model.	**5**
**Supplementary Figure 4**	Multiparameter Fluorescence Detection histograms.	**6**
**Supplementary Figure 5**	Dynamic averaging observed by time window analysis.	**8**
**Supplementary Figure 6**	Energy landscape of PDZ1-2 tandem from DMD simulations.	**10**
**Supplementary Figure 7**	Replica Exchange DMD simulation trajectories.	**12**
**Supplementary Figure 8**	Two dimensional PMF.	**13**
**Supplementary Figure 9**	Comparison of the interdye distributions as derived from DMD simulation ($p(R_{DA})_{PMF} = exp\left(-\frac{E_{PMF}}{0.6}\right)$) and the distances derived from MFD.	**14**
**Supplementary Figure 10**	Distance comparison between experiment and simulations of ensemble representatives for the open-like (OL) and closed-like (CL) states.	**16**
**Supplementary Figure 11**	Uncropped Scans of SDS-PAGE gels used for Disulfide mapping of the interdomain contact interface in the PDZ tandem.	**17**
**Supplementary Figure 12**	Engineered electro-negative and electropositive regions on PDZ domains.	**18**
**Supplementary Table 1**	Time Resolved fit results.<br>A) Donor Acceptor Distances and fractions.<br>B) Donor only Lifetime Decay.<br>C) Acceptor Only Lifetime Decay.	**19**
**Supplementary Table 2**	Time Resolved Anisotropy fit results<br>A) Fit results of the time resolved DOnly anisotropy.<br>B) Fit results of the time resolved acceptor anisotropy from direct excitation of the acceptor.<br>C) Fit results of the time resolved acceptor anisotropy sensitized by FRET.	**21**
**Supplementary Table 3**	Static FRET lines	**22**
**Supplementary Table 4**	Dynamic FRET Lines	**23**
**Supplementary Methods**	• *Ensemble Time Correlated Single Photon Counting (eTCSPC) analysis*<br>• *Accessible Volume (AV) simulations to estimate measured distance*<br>• *Analysis of Multiparameter Fluorescence Detection (MFD) for single-molecule FRET (smFRET) experiments*<br>• *Calculation of the Static and Dynamic FRET Lines*<br>• *Determination of Quantum Yields*<br>• *Estimation of $\langle\kappa^2\rangle$ and κ^2-distributions along with the associated uncertainty*	**25**

	• *Error propagation*<br>• *Global Analysis and goodness of fit.*	

Supplementary Figures

Supplementary Figure 1

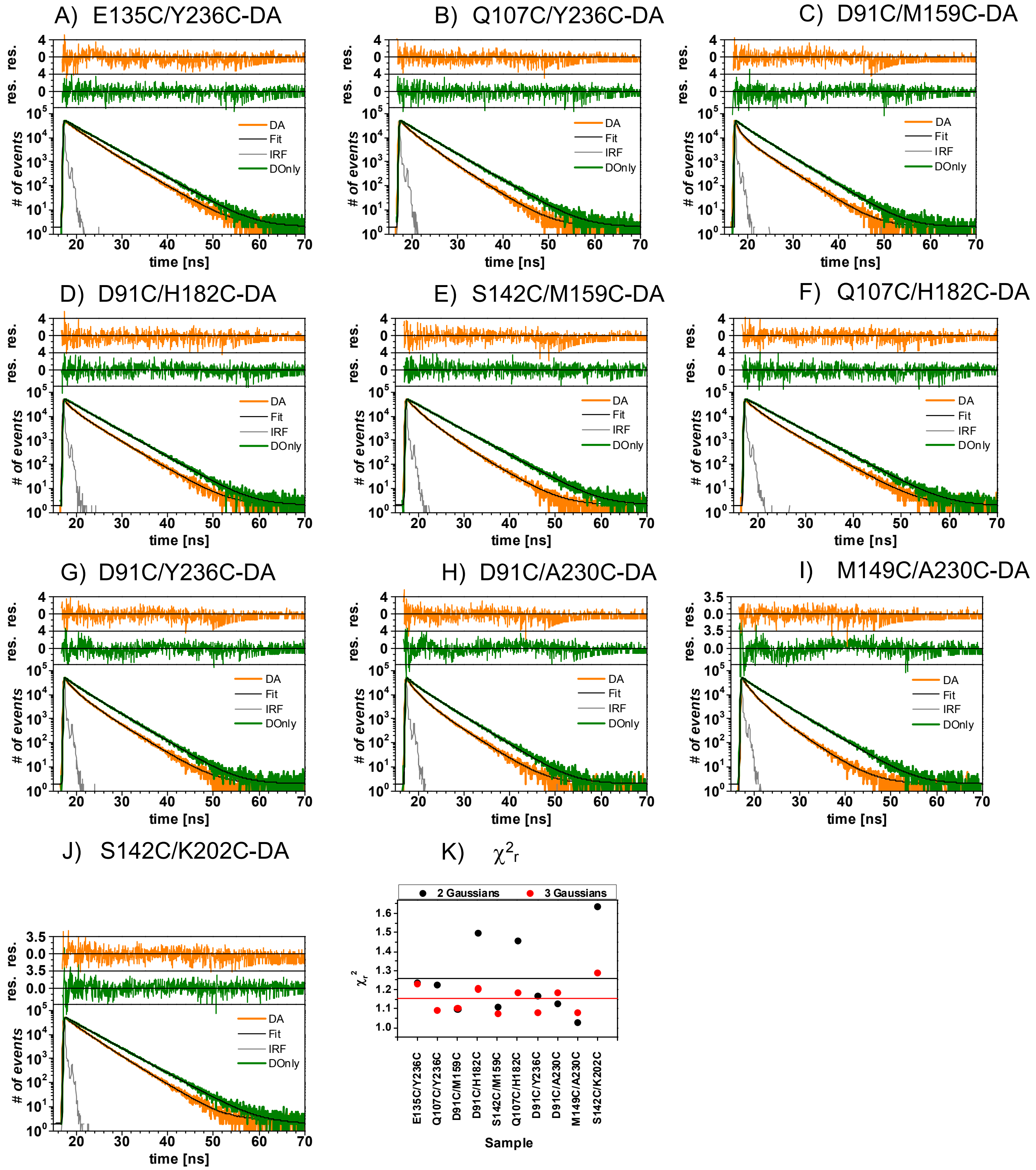

Supplementary Figure 1 Time Resolved Fluorescence of donor only (DOnly) and donor-acceptor (DA) labeled PDZ1-2 tandems. DOnly decays shown in green, and corresponding black overlay uses the Eq. 1. DA decays are shown in orange and model fit function uses Eqs. 3 and 4 to describe the Gaussian distribution of states. Instrument response function (IRF) is shown in gray. A) E135C/Y236C-DA B) Q107C/Y236C-DA C) D91C/M159C-DA D) D91C/H182C-DA, E) S142C/M159C-DA, F) Q107C/H182C-DA, G) D91C/Y236C-DA, H) D91C/A230C-DA, I) M149C/A230C-DA, J) S142C/K202C-DA. K) χ^2 comparison of two-Gaussian distributed states and three-Gaussian distributed states. Supplementary Table 1 summarizes the fit results.

Supplementary Figure 2

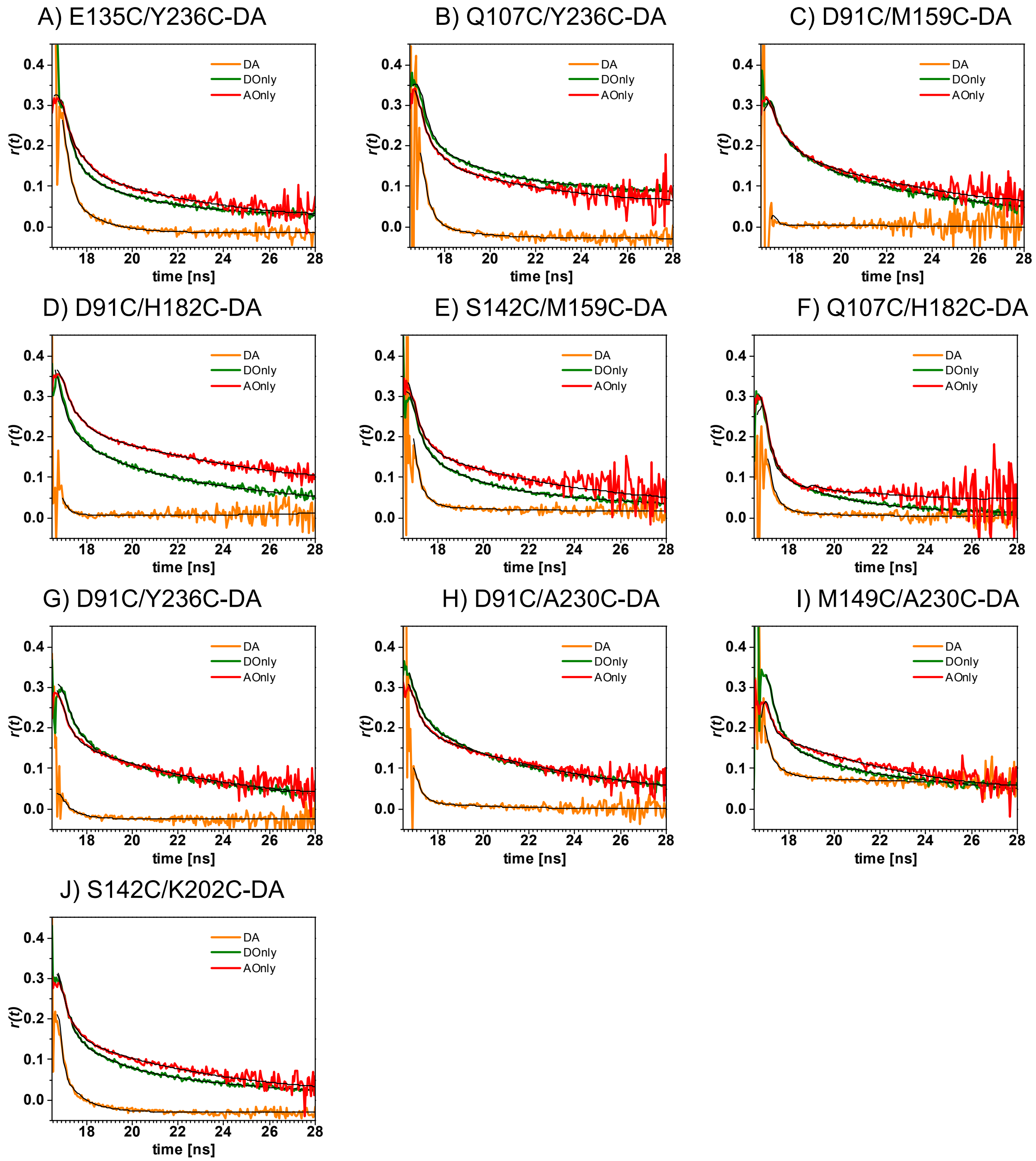

Supplementary Figure 2 Time Resolved Fluorescence Anisotropy of donor only (DOnly) and donor-acceptor (DA) labeled PDZ1-2 tandems. DOnly decays shown in green and corresponding black overlay use the Eqs. 1 and 6. DA decays are shown in orange and model fit function use Eqs. 4 and 6. Direct excitation of acceptor Instrument response function (IRF) is shown in gray. A) E135C/Y236C-DA B) Q107C/Y236C-DA C) D91C/M159C-DA D) D91C/H182C-DA, E) S142C/M159C-DA, F) Q107C/H182C-DA, G) D91C/Y236C-DA, H) D91C/A230C-DA, I) M149C/A230C-DA, J) S142C/K202C-DA. Supplementary Table 1 summarizes the fit results.

Supplementary Figure 3

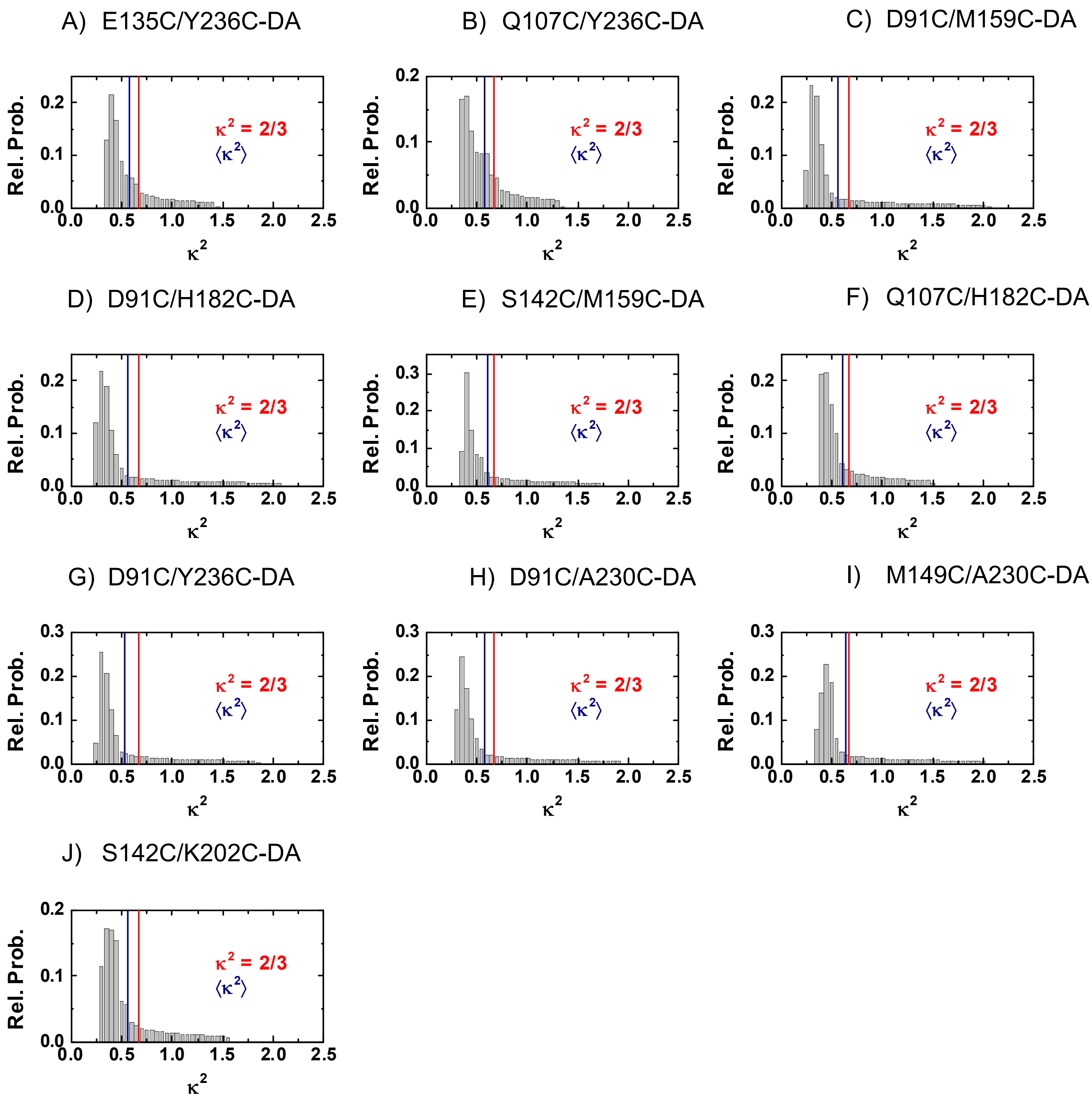

Supplementary Figure 3 κ^2 distribution using wobble in a cone model. A) E135C/Y236C-DA B) Q107C/Y236C-DA C) D91C/M159C-DA D) D91C/H182C-DA, E) S142C/M159C-DA, F) Q107C/H182C-DA, G) D91C/Y236C-DA, H) D91C/A230C-DA, I) M149C/A230C-DA, J) S142C/K202C-DA. Supplementary Table 1 summarizes the fit results. Red and blue vertical lines are the assumed value of 2/3 and the average value of the distribution, respectively. Distributions were obtained using time resolved anisotropy decays following Eqs. 12-14

Supplementary Figure 4

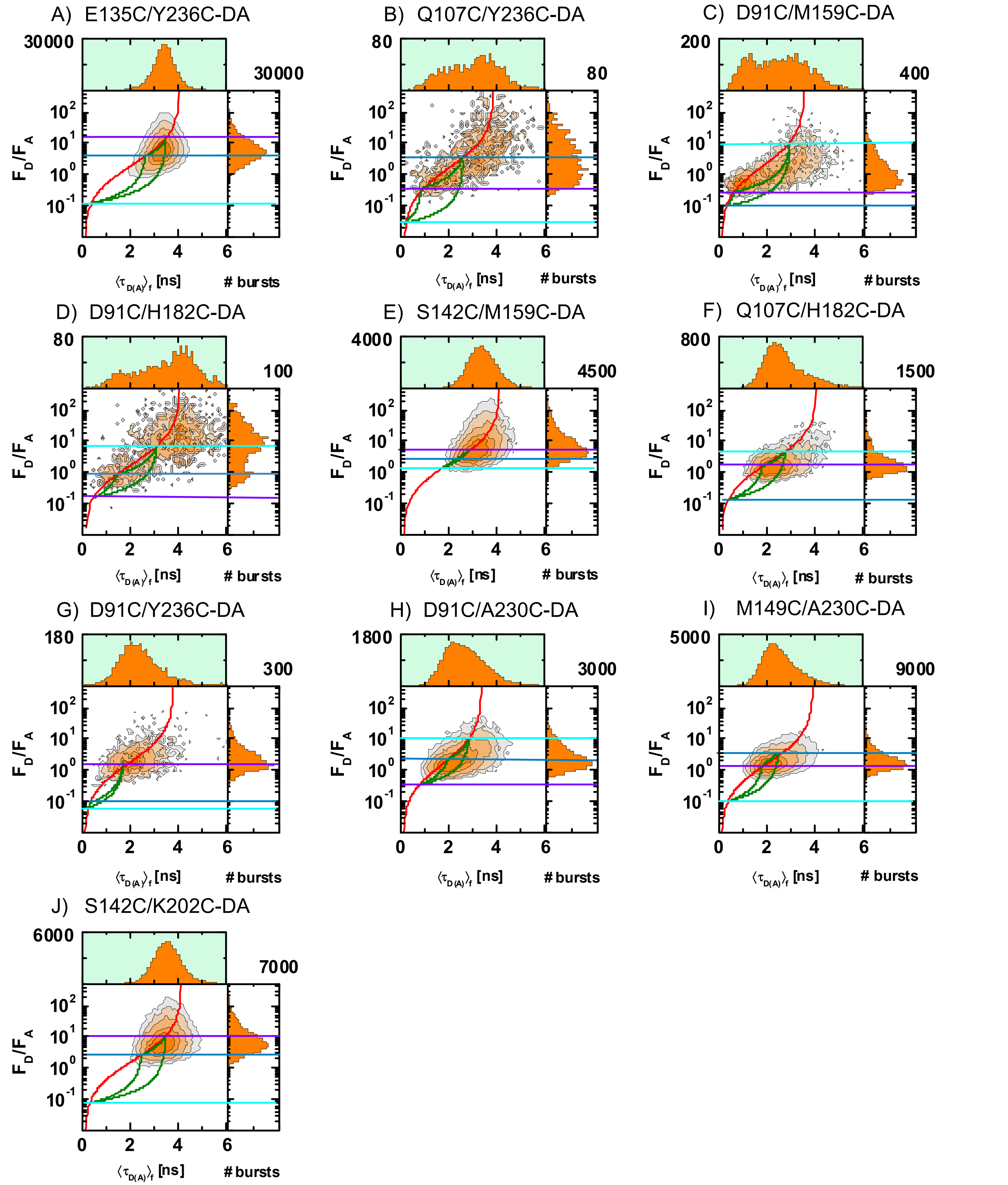

Supplementary Figure 4 Multiparameter Fluorescence Detection histograms. Each panel plots two FRET indicators for each single molecule event. The donor over acceptor fluorescence ratio (F_D/F_A) on the y-axis and

the average fluorescence lifetime ($\langle\tau_{D(A)}\rangle_f$) on the x-axis. Shown are the 2D histograms (orange contours) for A) E135C/Y236C-DA B) Q107C/Y236C-DA C) D91C/M159C-DA D) D91C/H182C-DA, E) S142C/M159C-DA, F) Q107C/H182C-DA, G) D91C/Y236C-DA, H) D91C/A230C-DA, I) M149C/A230C-DA, J) S142C/K202C-DA. Supplementary Table 1 summarizes the fit results. Horizontal guidelines for the major and minor states 1 and 2 are shown in purple and blue, respectively. Red and green lines correspond to the static and dynamic FRET lines (Supplementary Tables 3, 4), respectively.

Supplementary Figure 5

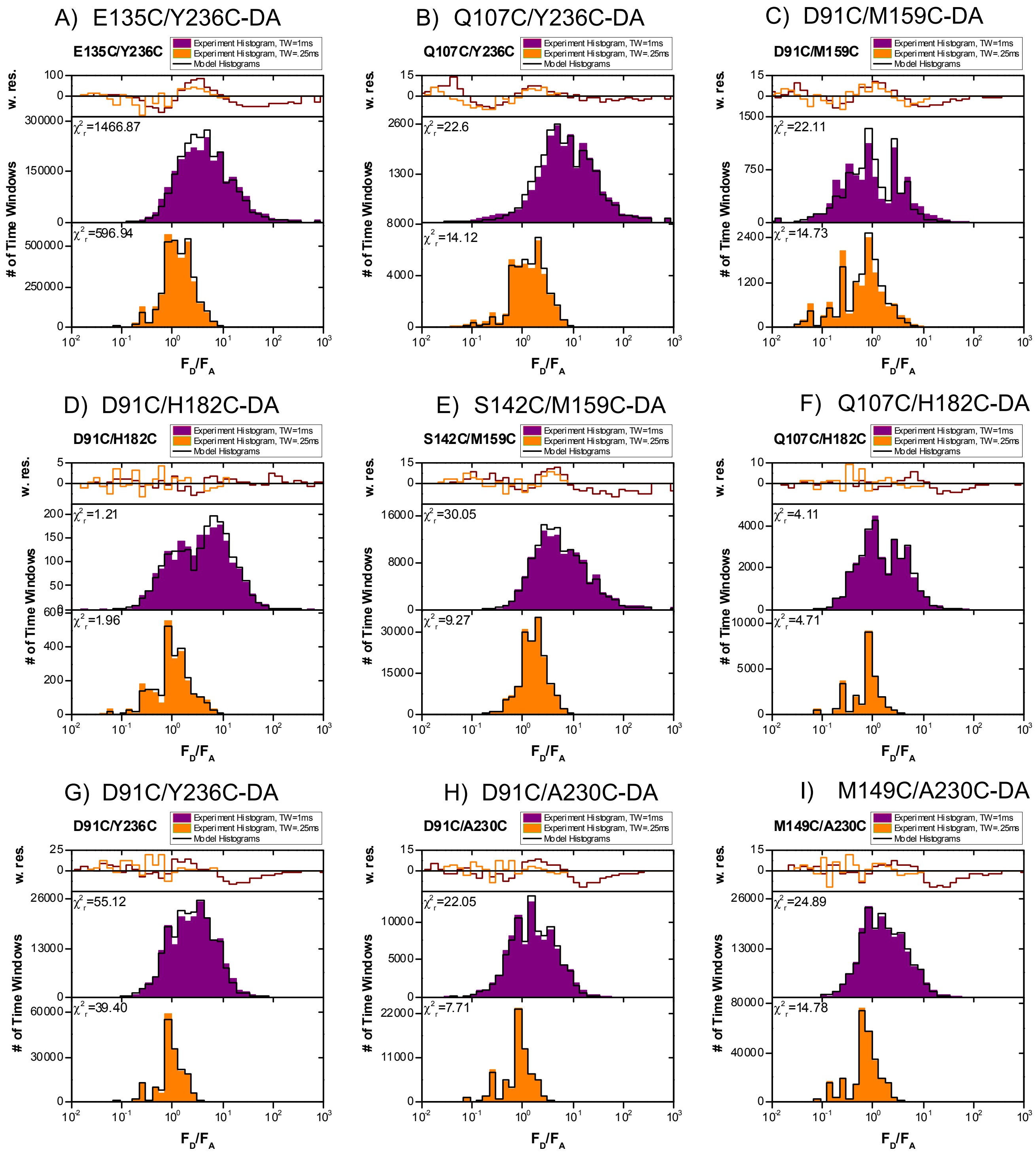

J) S142C/K202C-DA

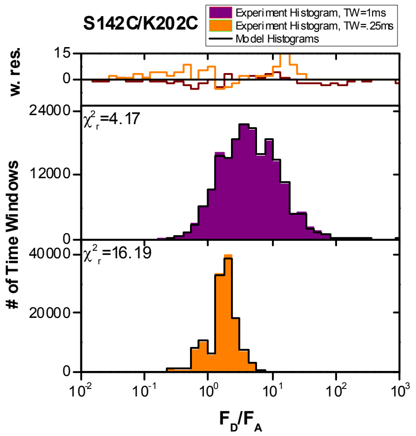

Supplementary Figure 5 Dynamic averaging observed by time window analysis. Two time windows (0.25 ms and 1 ms) were globally fit for each FRET variant. A) E135C/Y236C-DA B) Q107C/Y236C-DA C) D91C/M159C-DA D) D91C/H182C-DA, E) S142C/M159C-DA, F) Q107C/H182C-DA, G) D91C/Y236C-DA, H) D91C/A230C-DA, I) M149C/A230C-DA, J) S142C/K202C-DA. Each time window has its corresponding χ^2_r, which would not vary significantly between selected time windows for static molecules. The model used considers a single Gaussian distributed interdye distance with free standard deviation. The fit quality is poor indicating that a single state does not describe the system. Moreover, the figure of merit χ^2_r changes between time windows as indicative of dynamic effects.

Supplementary Figure 6

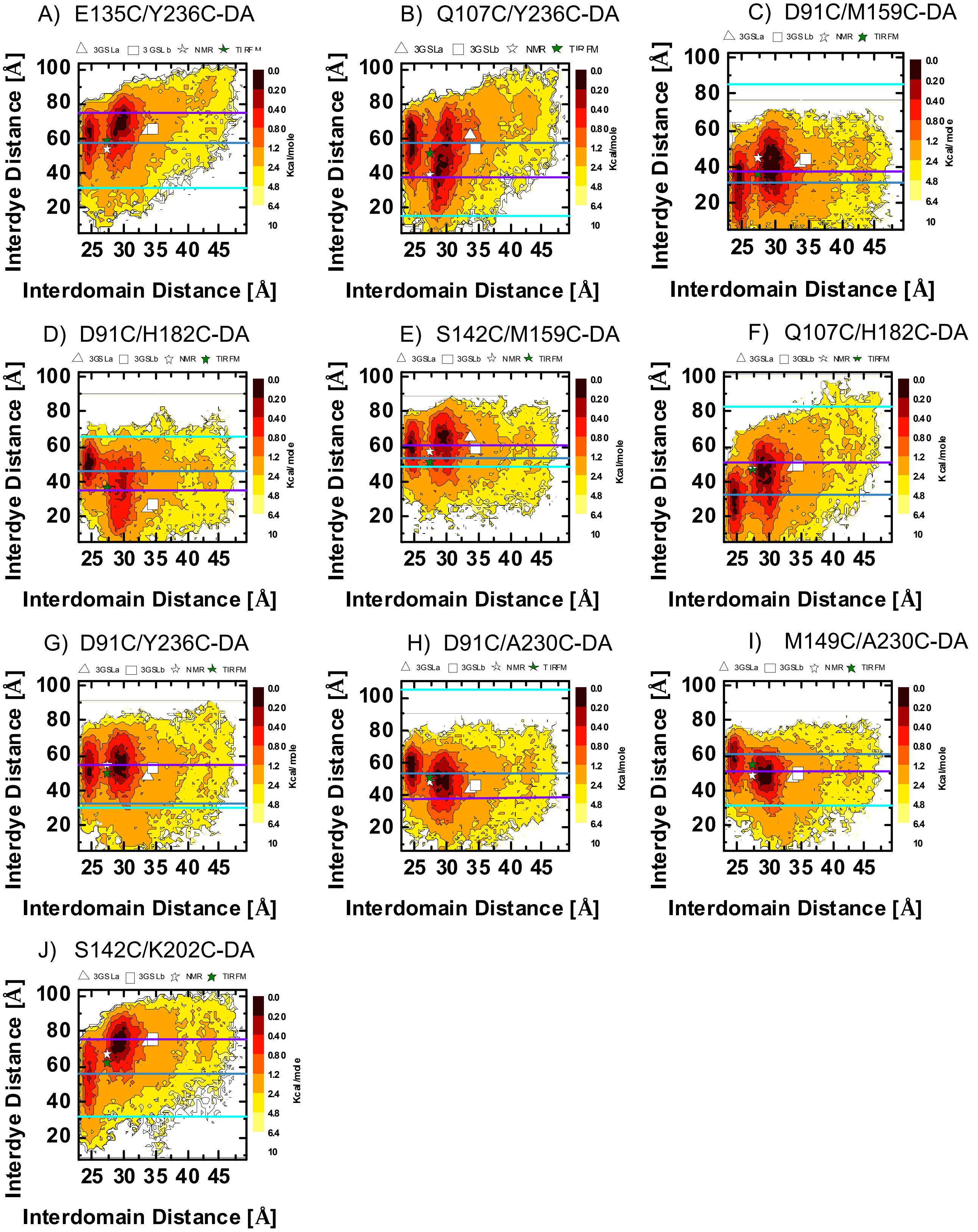

Supplementary Figure 6 Energy landscape of PDZ1-2 tandem from DMD simulations. A) E135C/Y236C-DA B) Q107C/Y236C-DA C) D91C/M159C-DA D) D91C/H182C-DA, E) S142C/M159C-DA, F) Q107C/H182C-DA, G) D91C/Y236C-DA, H) D91C/A230C-DA, I) M149C/A230C-DA, J) S142C/K202C-DA. Supplementary

Table 1 summarizes the fit results. Horizontal guidelines for the mean interdye states determined by eTCSPC are shown in purple and blue, respectively. Symbols represent the X-ray, NMR and TIRFM structural models.

Supplementary Figure 7

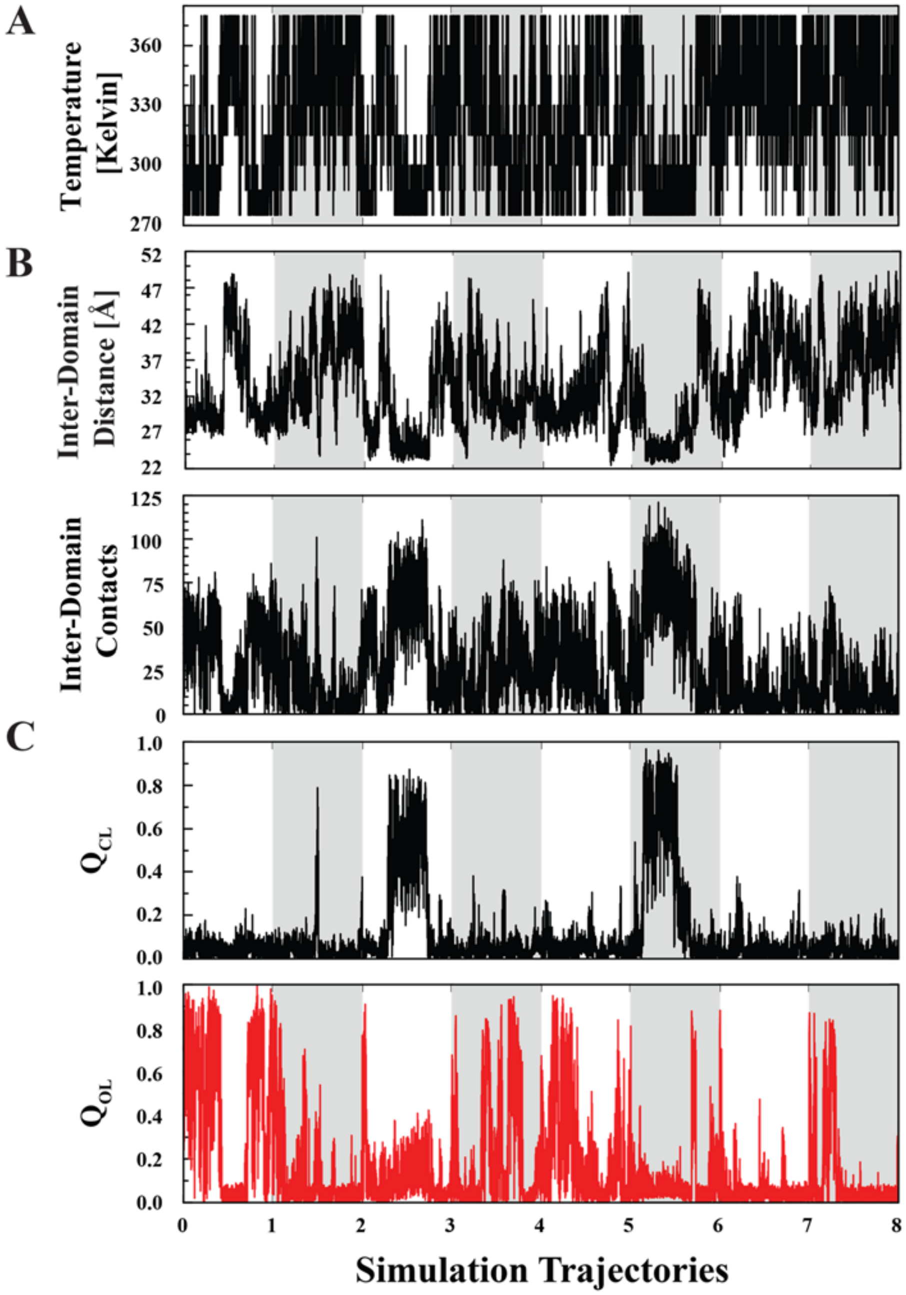

Supplementary Figure 7 Replica Exchange DMD simulation trajectories. A) Temperature as a function of the simulation time represented in terms of the replica or trajectory, B) Time evolution of the center of mass distance between two domains (Inter-Domain distance) and the number of Inter-Domain contacts between residues. Two residues are in contact if they have a least one atomic contact with a cutoff of 6.5 Å. C) The simulation trajectories of eight replicas in terms of the fraction of native contacts for the CL (closed-like) state (Q_{CL}) and the OL (open-like) state (Q_{OL}). Q-values for each state were computed according to native contact determined from corresponding structural ensembles (Fig. 4, and Supplementary Fig. 8). The total time for each replica is 200 ns, and there are 8 trajectories (0-7). The shading is used to separate different replicas.

Supplementary Figure 8

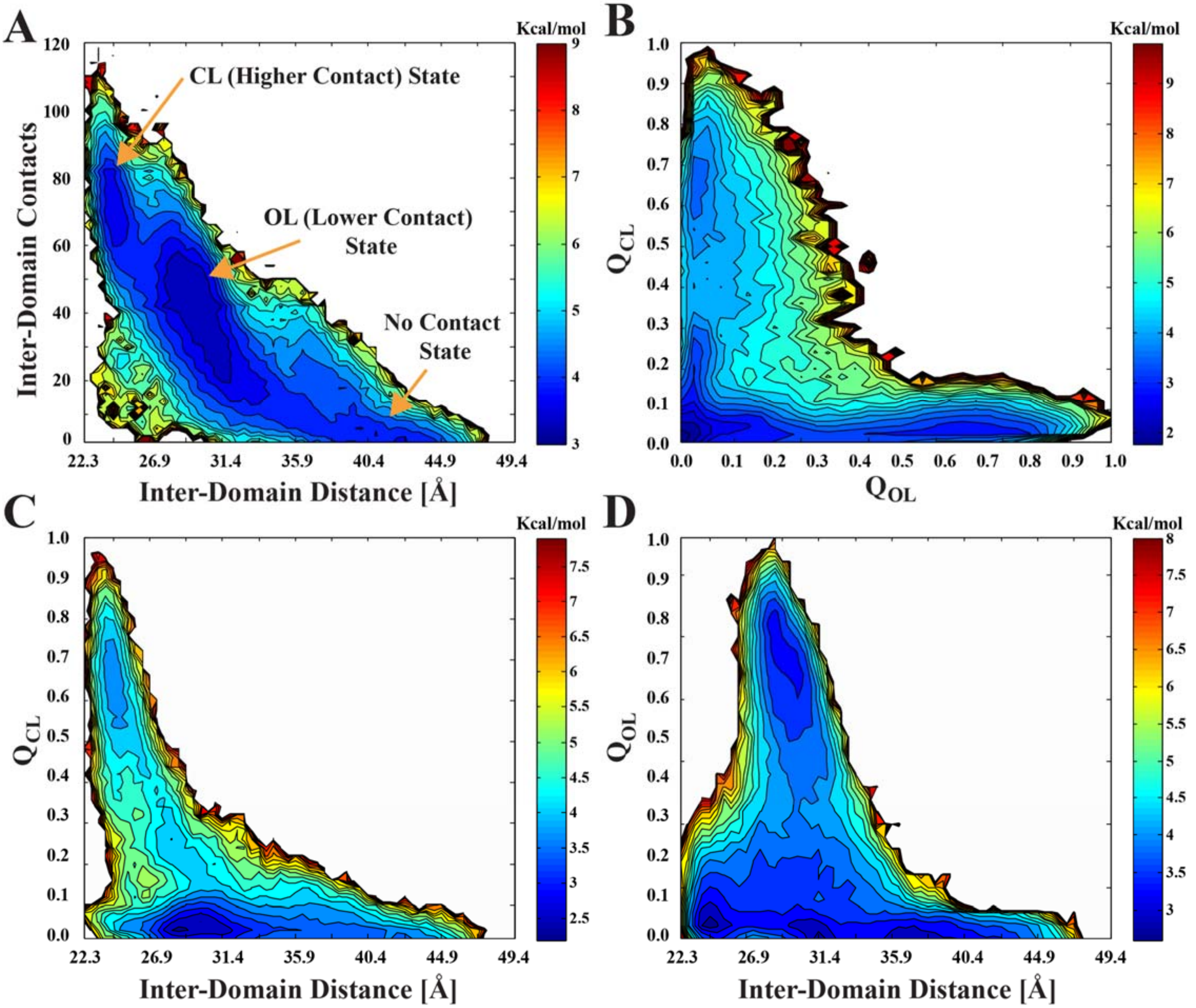

Supplementary Figure 8 Two dimensional PMF. The conformational dynamics of PDZ1-2 depicted by two-dimensional PMF (2D-PMF) plots projected onto different reaction coordinates, including the number of residue-wise contacts between two PDZ domains (Inter-Domain Contacts), the center of mass distance between two domains (Inter-Domain distance), and fractions of native contacts for the closed-like (CL) (Q_{CL}) and the open-like state (Q_{OL}). (A) From the PMF plot of Inter-Domain Contacts versus Inter-Domain Distance, two energy basins corresponding to the CL state and the OL state can be identified. (B) The 2D-PMF as the function of Q_{CL} and Q_{OL} suggests that these two states are exclusive to each other with different interface contacts (see also Fig. 4C,D) and the tandem domain in other state has to break all contacts in order to fold to the other state. The 2D-PMF as functions of (C) Q_{CL} versus Inter-domain and (D) Q_{OL} versus Inter-domain also highlight the separation of two states. The color bar denotes free energy scale in a unit of Kcal/mol.

Supplementary Figure 9

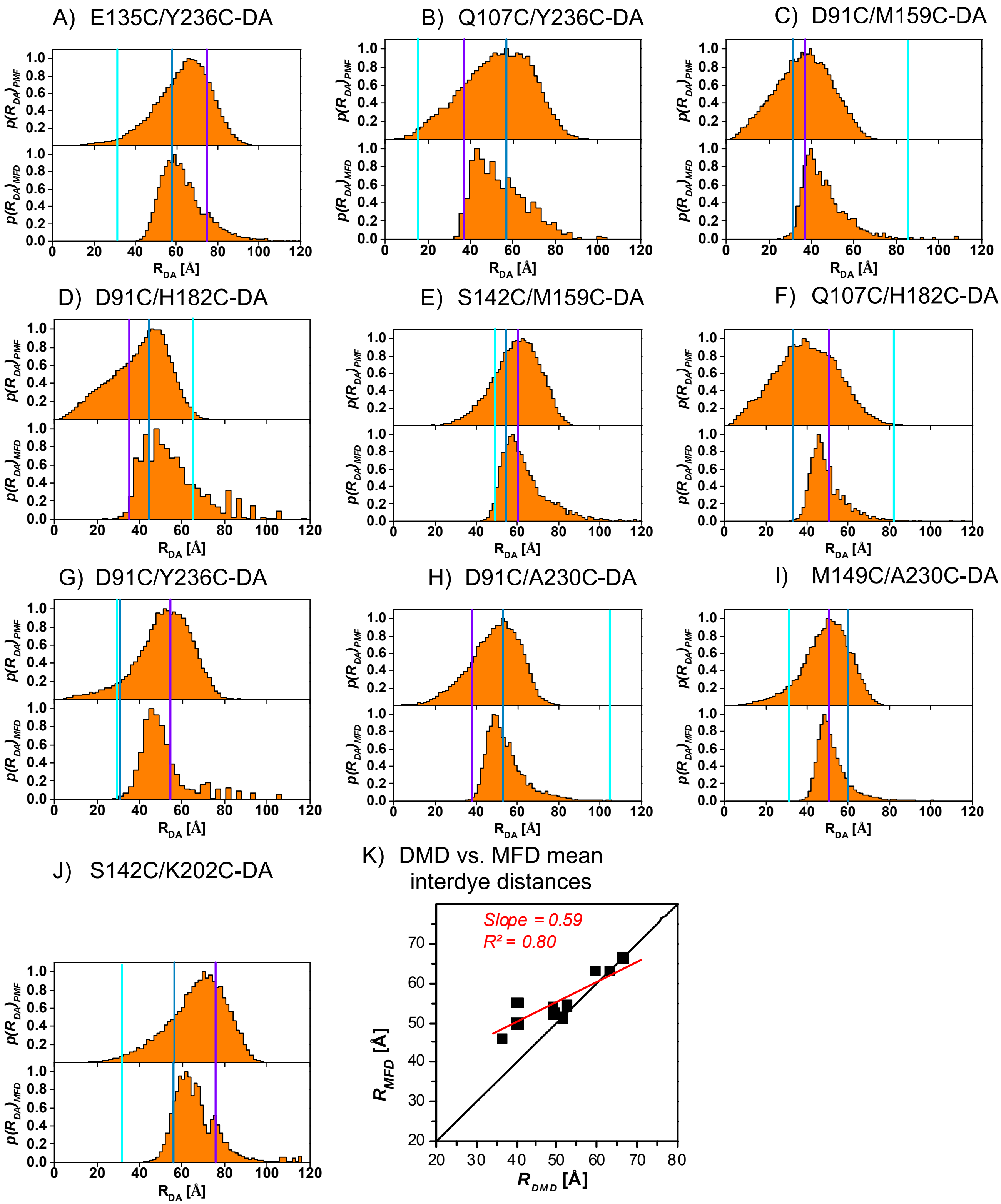

Supplementary Figure 9 Comparison of the interdye distributions as derived from DMD simulation ($p(R_{DA})_{PMF} = exp\left(-\frac{E_{PMF}}{0.6}\right)$**) and the distances derived from MFD**. A) E135C/Y236C-DA B) Q107C/Y236C-DA C) D91C/M159C-DA D) D91C/H182C-DA, E) S142C/M159C-DA, F) Q107C/H182C-DA, G) D91C/Y236C-DA, H) D91C/A230C-DA, I) M149C/A230C-DA, J) S142C/K202C-DA. F_D/F_A distributions are converted into

interdye distances following $R_{DA} = R_0 \left(\frac{\Phi_{FA}}{\Phi_{FD(0)}} \cdot \frac{F_D}{F_A} \right)^{1/6}$ [12]. K) Correlation between the mean interdye distances as derived for each variant using DMD interdye distributions and mean interdye distances derived from MFD distance distributions.

Supplementary Figure 10

A) Two-Gaussian distributed states

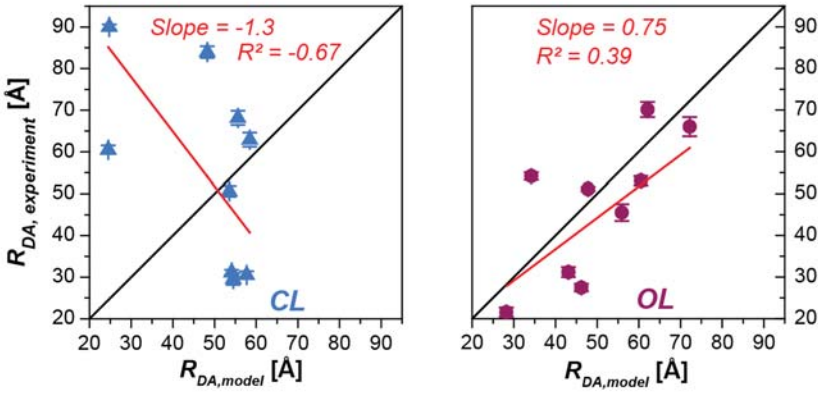

B) Three-Gaussian distributed states

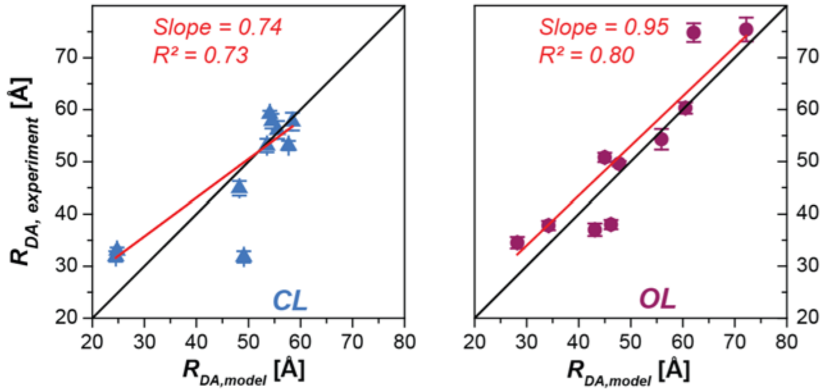

C) Overlay of the two representative structures

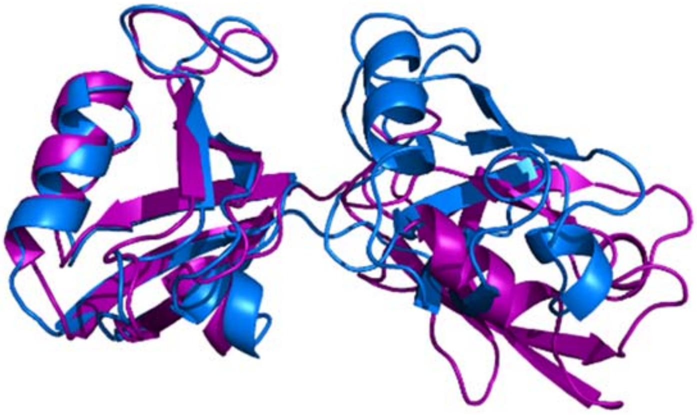

Supplementary Figure 10 Distance comparison between experiment and simulations of ensemble representatives for the open-like (OL) and closed-like (CL) states. A) Using a two-Gaussian distributed state model and B) with a three-Gaussian distributed state. Error bars on panel A and B correspond to uncertainties on the measured interdye distance. C) Cartoon representation of the two representative structures (Fig. 4A-B) overlaying PDZ1. The significant rotation of PDZ2 with respect of PDZ1 is observed. Blue is the CL state and magenta corresponds to the OL state.

Supplementary Figure 11

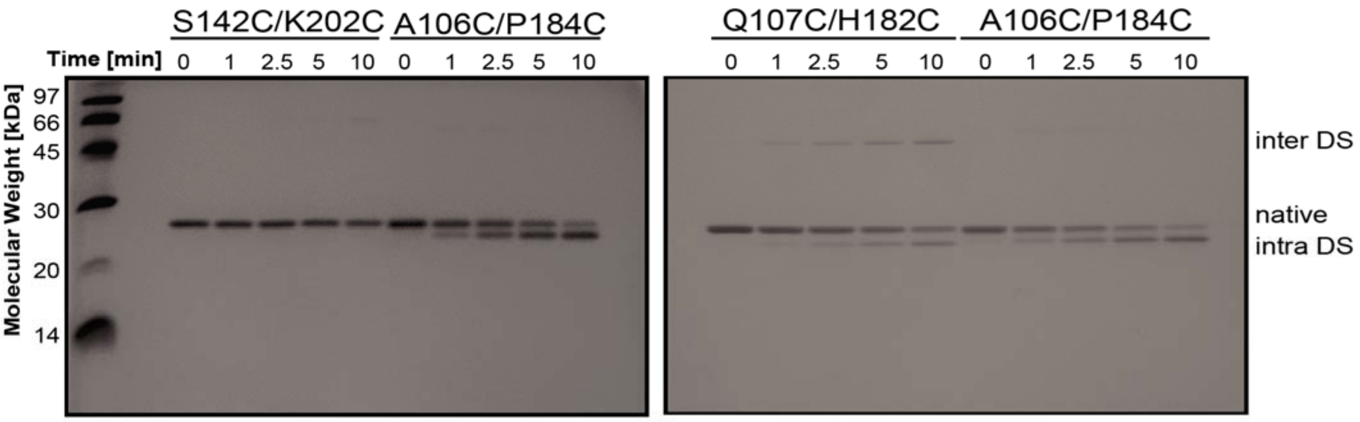

Supplementary Figure 11 Uncropped Scans of SDS-PAGE gels used for Disulfide mapping of the interdomain contact interface in the PDZ tandem. A cartoon representation of the location of engineered disulfides is shown in Fig. 5 in the main text. Representative SDS-PAGE showing individual time points used for the analysis of the extent and kinetics of the disulfide bond formation reactions. Disulfide formation was initiated by the addition of 0.5 mM $CuSO_4$ and 1.75 mM 1, 10-phenanthroline. Time points were quenched by adding 40 mM N-ethylmaleimide to alkylate unbonded cysteines and 10 mM EDTA followed immediately by boiling at 95 °C for 5 minutes in non-reducing Laemmli sample buffer. The location of the introduced cysteine residues for each mutant is labeled above the panels. Duration of the reaction for each time point is indicated above each lane. The molecular weights of the standards (GE Healthcare) are indicated to the left of the panels. The position of the native PDZ Tandem along with the intramolecular and intermolecular disulfide products are indicated to the right of the panels. Reactions were run in triplicate to obtain the percentage of disulfide for each time point. Each time course was fit with an exponential function to obtain the initial and final percentage of disulfide bonding along with the rate of disulfide formation as shown in Fig. 5.

Supplementary Figure 12

A)

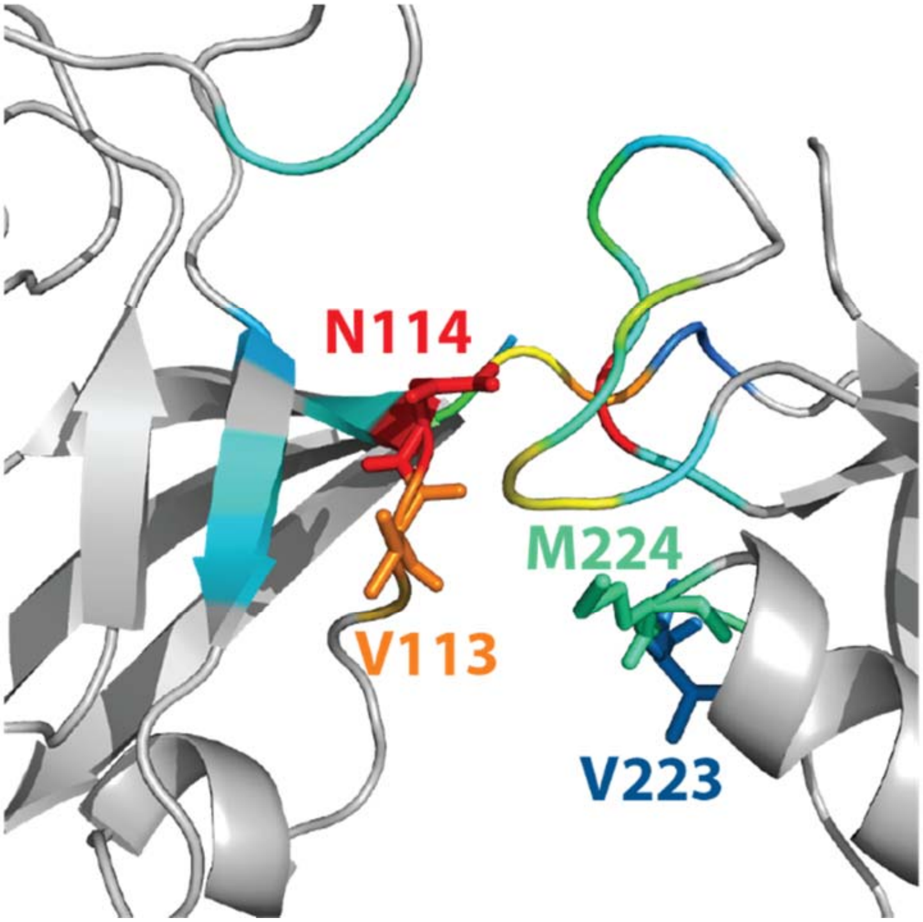

B) F_D/F_A Histogram

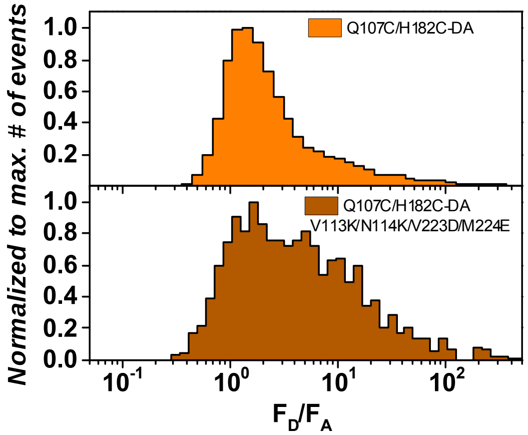

C) Time Resolved Fluorescence Decay

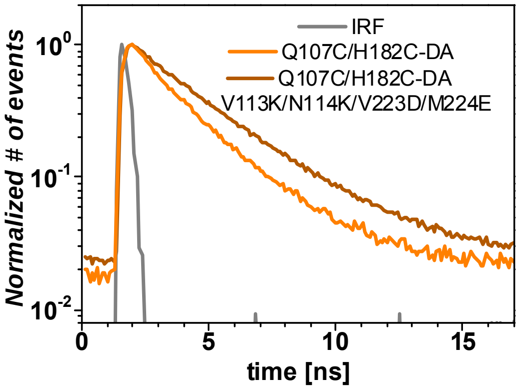

Supplementary Figure 12 Engineered electro-negative and electropositive regions on PDZ domains. A) Cartoon representation of the open-like state showing the residues that were selected for mutations (V113K/N114K/V223D/M224E) B) F_D/F_A Histogram of the Q107C/H182C (**Orange**) and the engineered salt bridge V113K/N114K/V223D/M224E (**brown**). This last one shifts the equilibrium towards lower FRET efficiencies or higher F_D/F_A ratio. C) Time Resolved Fluorescence of the Donor emission in the presence of acceptor for the Q107C/H182C and the engineered salt bridge V113K/N114K/V223D/M224E. The mutated interface shows a slower decay, consistent with the F_D/F_A ratio. The shift towards lower FRET is consistent

with the anticipated clockwise motion of PDZ2 (right domain in panel A) with respect to PDZ1 (left domain in panel A), which in turn it will push farther the labels located at Q107C and H182C to center the engineered interface.

Supplementary Tables

Supplementary Table 1 Time Resolved fit results.

A) Donor Acceptor Distances and fractions as model with a three Gaussian distributed and the addition of a No FRET state (Eq. 4). There is a major population representing the 43.5%. The second most populated accounts for 32.4%, and the least populated corresponds to 24.1%.

Sample	σ_{1DA} [Å]	$\langle R_{DA}\rangle_1 \pm \varepsilon$ [Å]	σ_{2DA} [Å]	$\langle R_{DA}\rangle_2 \pm \varepsilon$ [Å]	σ_{3DA} [Å]	$\langle R_{DA}\rangle_3 \pm \varepsilon$ [Å]	No FRET	χ_r^2
**E135C/Y236C-DA**	2.6	74.8 ± 1.8	8.7	57.7 ± 1.7	5.8	32.6 ± 0.8	0.28	1.233
**Q107C/Y236C-DA**	1.3	37.8 ± 0.9	8.4	57.8 ± 1.4	0.7	15.7 ± 1.1	0.15	1.095
**D91C/M159C-DA**	9.1	37.0 ± 1.2	5.0	31.9 ±1.0	3.0	85.2 ± 2.6	0.09	1.104
**D91C/H182C-DA**	8.0	34.5 ± 1.1	6.1	44.9 ± 1.4	8.2	65.7 ± 2.1	0.21	1.206
**S142C/M159C-DA**	0.5	60.3 ± 1.1	3.4	53.1 ± 0.9	13.9	47.8 ± 0.8	0.09	1.078
**Q107C/H182C-DA**	4.4	50.8 ± 0.9	4.5	33.0 ± 0.6	4.8	81.7 ± 1.4	0.12	1.183
**D91C/Y236C-DA**	3.9	54.3 ± 2.0	15.5	31.8 ± 1.1	2.8	29.7 ± 1.1	0.22	1.083
**D91C/A230C-DA**	10.6	38.0 ± 0.9	3.7	53.1 ± 1.3	2.7	105.7 ± 2.7	0.11	1.183
**M149C/A230C-DA**	3.3	49.6 ± 0.5	2.3	59.2 ± 0.6	11.1	31.8 ± 0.3	0.07	1.078
**S142C/K202C-DA**	5.7	75.4 ± 2.3	4.5	56.1 ± 1.7	1.9	31.2 ± 1.0	0.00	1.290

B) Donor only Lifetime Decay. $\Phi_{FD(0)}$ determination is described in methods section. Goodness of fit evaluated for 1 (χ_{r1}^2) and 2 (χ_{r2}^2) exponential decays.

Sample	x_1	τ_1 [ns]	x_2	τ_2 [ns]	$\langle\tau\rangle_x$ [ns]	$\langle\tau\rangle_f$ [ns]	$\Phi_{D(0)}$	χ_{r2}^2	χ_{r1}^2
**E135C/Y236C-DA**	0.92	4.13	0.08	1.24	3.90	4.06	0.78	1.172	2.613
**Q107C/Y236C-DA**	0.87	3.99	0.13	1.46	3.65	3.85	0.73	1.288	4.091
**D91C/M159C-DA**	0.77	3.76	0.23	1.17	3.16	3.54	0.63	1.386	12.53
**D91C/H182C-DA**	0.92	4.10	0.08	1.10	3.86	4.03	0.77	1.361	2.448
**S142C/M159C-DA**	0.90	4.24	0.10	1.54	3.98	4.14	0.80	1.343	2.791
**Q107C/H182C-DA**	0.94	4.14	0.06	1.15	2.85	4.09	0.79	1.295	2.020
**D91C/Y236C-DA**	0.74	3.29	0.26	1.03	2.71	3.07	0.54	1.556	14.98
**D91C/A230C-DA**	0.66	3.72	0.34	1.15	2.85	3.37	0.57	1.473	26.35
**M149C/A230C-DA**	0.76	3.85	0.24	1.13	3.20	3.62	0.64	1.711	13.89
**S142C/K202C-DA**	0.89	4.22	0.11	1.05	3.86	4.12	0.77	1.674	4.384

C) Acceptor Only Lifetime Decay. Φ_{FA} determination is described in methods section

Sample	x_1	τ_1	x_2	τ_2	$\langle\tau\rangle_x$ [ns]	$\langle\tau\rangle_f$ [ns]	Φ_{FA}	χ_r^2
**E135C/Y236C-DA**	0.68	1.54	0.32	0.77	1.30	1.40	0.35	1.174
**Q107C/Y236C-DA**	0.48	1.71	0.52	1.19	1.44	1.49	0.39	1.748
**D91C/M159C-DA**	0.60	1.65	0.40	0.72	1.28	1.44	0.35	1.849
**D91C/H182C-DA**	0.66	1.73	0.34	0.72	1.38	1.55	0.38	1.815
**S142C/M159C-DA**	0.58	1.53	0.42	0.86	1.25	1.34	0.34	1.447

**Q107C/H182C-DA**	0.59	1.57	0.41	0.67	1.20	1.36	0.33	0.885
**D91C/Y236C-DA**	0.65	1.69	0.35	0.86	1.40	1.51	0.38	1.643
**D91C/A230C-DA**	0.62	1.83	0.38	0.93	1.49	1.62	0.41	1.423
**M149C/A230C-DA**	0.66	1.79	0.34	0.90	1.49	1.61	0.41	1.413
**S142C/K202C-DA**	0.60	1.91	0.40	1.00	1.54	1.67	0.42	1.423

Supplementary Table 2 Time Resolved Anisotropy.

A) Fit results of the time resolved DOnly anisotropy.

Sample	b_1	ρ_1 [ns]	b_2	ρ_2 [ns]	b_3	ρ_3 [ns]	$\chi^2_{r,sum}$	$\chi^2_{r,diff}$	r_{ss} +/- std
**E135C/Y236C-DA**	0.12	0.33	0.09	1.99	0.06	12.24	2.86	1.03	0.1 ±0.01
**Q107C/Y236C-DA**	0.16	0.31	0.11	2.32	0.12	37.00	2.90	1.14	0.2 ±0.00
**D91C/M159C-DA**	0.10	0.22	0.09	1.27	0.17	8.81	1.69	1.10	0.2±0.00
**D91C/H182C-DA**	0.12	0.35	0.08	1.91	0.15	10.97	2.59	0.95	0.2 ±0.00
**S142C/M159C-DA**	0.15	0.29	0.10	2.06	0.08	14.00	4.40	1.51	0.1 ±0.02
**Q107C/H182C-DA**	0.25	0.22	0.10	2.63	0.03	13.49	4.17	1.51	0.1 ±0.00
**D91C/Y236C-DA**	0.14	0.62			0.16	7.39	2.23	1.16	0.1 ±0.01
**D91C/A230C-DA**	0.11	0.35	0.12	2.89	0.12	15.47	3.42	1.28	0.2 ±0.01
**M149C/A230C-DA**	0.17	0.36	0.07	2.59	0.10	14.94	3.51	1.32	0.1 ±0.02
**S142C/K202C-DA**	0.14	0.27	0.09	1.45	0.10	7.91	3.68	1.33	0.1 ±0.01

B) Fit results of the time resolved acceptor anisotropy from direct excitation of the acceptor.

Sample	b_1	ρ_1 [ns]	b_2	ρ_2 [ns]	b_3	ρ_3 [ns]	$\chi^2_{r,sum}$	$\chi^2_{r,diff}$	r_{ss} +/- std
**E135C/Y236C-DA**	0.19	0.58	0.15	6.94			9.28	1.74	0.2 ±0.01
**Q107C/Y236C-DA**	0.17	0.48	0.12	6.04	0.05	5691	6.55	1.36	0.2 ±0.06
**D91C/M159C-DA**	0.14	0.64	0.19	9.83			6.14	1.33	0.2 ±0.02
**D91C/H182C-DA**	0.15	0.56	0.22	13.79			31.58	4.52	0.2 ±0.02
**S142C/M159C-DA**	0.15	0.44	0.17	8.81			2.78	1.30	0.2 ±0.03
**Q107C/H182C-DA**	0.26	0.43	0.08	18.24			3.17	1.18	0.1 ±0.01
**D91C/Y236C-DA**	0.12	0.41	0.17	7.46			3.16	1.29	0.2 ±0.03
**D91C/A230C-DA**	0.11	0.39	0.19	8.94			3.52	1.11	0.2 ±0.02
**M149C/A230C-DA**	0.17	0.19	0.19	8.28			4.35	1.37	0.2 ±0.05
**S142C/K202C-DA**	0.14	0.43	0.16	6.97			5.03	1.82	0.2 ±0.04

C) Fit results of the time resolved acceptor anisotropy sensitized by FRET.

Sample	b_1	ρ_1 [ns]	b_2	ρ_2 [ns]	b_∞	ρ_∞ [ns]	$\chi^2_{r,sum}$	$\chi^2_{r,diff}$	r_{ss} +/- std
**E135C/Y236C-DA**	0.22	0.16	0.10	1.28	-0.01	285	3.31	1.10	0.0 ±0.03
**Q107C/Y236C-DA**	0.10	0.32	0.04	1.78	-0.03	61887	2.44	1.11	0.0 ±0.08
**D91C/M159C-DA**	0.003	0.31			0.005	15	1.79	1.08	0.0 ±0.03
**D91C/H182C-DA**	0.02	0.24			0.007	154633	1.84	1.07	0.0 ±0.02
**S142C/M159C-DA**	0.07	0.40			0.03	18	2.78	0.98	0.0 ±0.11
**Q107C/H182C-DA**	0.09	0.34			0.01	5	2.50	0.98	0.0 ±0.03
**D91C/Y236C-DA**	0.04	0.59			-0.03	59417	2.43	1.05	-0.0±0.07
**D91C/A230C-DA**	0.04	0.28			0.02	3	2.28	0.96	0.0 ±0.07
**M149C/A230C-DA**	0.06	0.51			0.08	45	1.98	1.01	0.1 ±0.14
**S142C/K202C-DA**	0.10	0.82			-0.03	164115	3.34	1.19	-0.0±0.08

Supplementary Table 3 Static FRET lines

Sample	*FRET Lines*
**E135C/Y236C-DA**	$(0.7798/0.35)/((4.0565/((-0.0381*\langle\tau_{D(A)}\rangle_f^3)+(0.2694*\langle\tau_{D(A)}\rangle_f^2)+0.5465*\langle\tau_{D(A)}\rangle_f +-0.0505))-1)$
**Q107C/Y236C-DA**	$(0.7718/0.39)/((3.8588/((-0.0497*\langle\tau_{D(A)}\rangle_f^3)+(0.3622*\langle\tau_{D(A)}\rangle_f^2)+0.3548*\langle\tau_{D(A)}\rangle_f +-0.0509))-1)$
**D91C/M159C-DA**	$(0.6329/0.35)/((3.5397/((-0.0546*\langle\tau_{D(A)}\rangle_f^3)+(0.2997*\langle\tau_{D(A)}\rangle_f^2)+0.6392*\langle\tau_{D(A)}\rangle_f +-0.0600))-1)$
**D91C/H182C-DA**	$(0.7720/0.38)/((4.0316/((-0.0386*\langle\tau_{D(A)}\rangle_f^3)+(0.2699*\langle\tau_{D(A)}\rangle_f^2)+0.5508*\langle\tau_{D(A)}\rangle_f +-0.0508))-1)$
**S142C/M159C-DA**	$(0.7940/0.34)/((4.1353/((-0.0370*\langle\tau_{D(A)}\rangle_f^3)+(0.2645*\langle\tau_{D(A)}\rangle_f^2)+0.5502*\langle\tau_{D(A)}\rangle_f +-0.0515))-1)$
**Q107C/H182C-DA**	$(0.7912/0.33)/((4.0832/((-0.0372*\langle\tau_{D(A)}\rangle_f^3)+(0.2681*\langle\tau_{D(A)}\rangle_f^2)+0.5364*\langle\tau_{D(A)}\rangle_f +-0.0492))-1)$
**D91C/Y236C-DA**	$(0.7578/0.38)/((3.7890/((-0.0424*\langle\tau_{D(A)}\rangle_f^3)+(0.2987*\langle\tau_{D(A)}\rangle_f^2)+0.4866*\langle\tau_{D(A)}\rangle_f +-0.0416))-1)$
**D91C/A230C-DA**	$(0.5692/0.41)/((3.3669/((-0.0621*\langle\tau_{D(A)}\rangle_f^3)+(0.2944*\langle\tau_{D(A)}\rangle_f^2)+0.7327*\langle\tau_{D(A)}\rangle_f +-0.0708))-1)$
**M149C/A230C-DA**	$(0.7802/0.41)/((3.9010/((-0.0397*\langle\tau_{D(A)}\rangle_f^3)+(0.2882*\langle\tau_{D(A)}\rangle_f^2)+0.4903*\langle\tau_{D(A)}\rangle_f +-0.0425))-1)$
**S142C/K202C-DA**	$(0.7743/0.42)/((4.1254/((-0.0371*\langle\tau_{D(A)}\rangle_f^3)+(0.2576*\langle\tau_{D(A)}\rangle_f^2)+0.5814*\langle\tau_{D(A)}\rangle_f +-0.0551))-1)$

Supplementary Table 4 Dynamic FRET Lines

Sample	*Transition States*	*Dynamic FRET Lines*
**E135C/Y236C-DA**	1-2	0.1922/(0.35*((1/2.6182+1/3.4779-(1.1192*$\langle\tau_{D(A)}\rangle_f$ +-0.4056)/(3.4779*2.6182))-1/4.0565))
	2-3	0.1922/(0.35*((1/0.4107+1/2.6180-(2.1957*$\langle\tau_{D(A)}\rangle_f$ +-3.1428)/(2.6180*0.4107))-1/4.0565))
	3-1	0.1922/(0.35*((1/3.4779+1/0.4110-(2.1484*$\langle\tau_{D(A)}\rangle_f$ +-3.9927)/(0.4110*3.4779))-1/4.0565))
**Q107C/Y236C-DA**	1-2	0.1898/(0.39*((1/2.5947+1/0.8567-(1.6334*$\langle\tau_{D(A)}\rangle_f$ +-1.6540)/(0.8567*2.5947))-1/3.8588))
	2-3	0.1898/(0.39*((1/0.1616+1/2.5950-(3.1465*$\langle\tau_{D(A)}\rangle_f$ +-5.5739)/(2.5950*0.1616))-1/3.8588))
	3-1	0.1898/(0.39*((1/0.8567+1/0.1620-(3.3811*$\langle\tau_{D(A)}\rangle_f$ +-2.1081)/(0.1620*0.8567))-1/3.8588))
**D91C/M159C-DA**	1-2	0.1788/(0.35*((1/0.3363+1/0.6581-(2.4540*$\langle\tau_{D(A)}\rangle_f$ +-1.1029)/(0.6581*0.3363))-1/3.5397))
	2-3	0.1788/(0.35*((1/2.9473+1/0.3360-(2.0567*$\langle\tau_{D(A)}\rangle_f$ +-3.1084)/(0.3360*2.9473))-1/3.5397))
	3-1	0.1788/(0.35*((1/0.6581+1/2.9470-(1.5663*$\langle\tau_{D(A)}\rangle_f$ +-1.6555)/(2.9470*0.6581))-1/3.5397))
**D91C/H182C-DA**	1-2	0.1915/(0.38*((1/1.4315+1/0.5166-(2.1498*$\langle\tau_{D(A)}\rangle_f$ +-1.7526)/(0.5166*1.4315))-1/4.0316))
	2-3	0.1915/(0.38*((1/3.0955+1/1.4310-(1.3838*$\langle\tau_{D(A)}\rangle_f$ +-1.1915)/(1.4310*3.0955))-1/4.0316))
	3-1	0.1915/(0.38*((1/0.5166+1/3.0950-(1.9710*$\langle\tau_{D(A)}\rangle_f$ +-3.0072)/(3.0950*0.5166))-1/4.0316))
**S142C/M159C-DA**	1-2	0.1920/(0.34*((1/2.2646+1/2.8450-(1.2376*$\langle\tau_{D(A)}\rangle_f$ +-0.7078)/(2.8450*2.2646))-1/4.1353))
	2-3	0.1920/(0.34*((1/1.7475+1/2.2650-(1.4044*$\langle\tau_{D(A)}\rangle_f$ +-1.0204)/(2.2650*1.7475))-1/4.1353))
	3-1	0.1920/(0.34*((1/2.8450+1/1.7470-(1.3365*$\langle\tau_{D(A)}\rangle_f$ +-0.9823)/(1.7470*2.8450))-1/4.1353))
**Q107C/H182C-DA**	1-2	0.1938/(0.33*((1/0.4287+1/1.8140-(2.2672*$\langle\tau_{D(A)}\rangle_f$ +-2.3565)/(1.8140*0.4287))-1/4.0832))
	2-3	0.1938/(0.33*((1/2.7692+1/0.4290-(2.1777*$\langle\tau_{D(A)}\rangle_f$ +-3.2727)/(0.4290*2.7692))-1/4.0879))
	3-1	0.1938/(0.33*((1/1.8140+1/2.7690-(1.3466*$\langle\tau_{D(A)}\rangle_f$ +-1.0030)/(2.7690*1.8140))-1/4.0879))
**D91C/Y236C-DA**	1-2	0.1763/(0.38*((1/0.3277+1/1.7191-(2.0601*$\langle\tau_{D(A)}\rangle_f$ +-1.8213)/(1.7191*0.3277))-1/3.0660))
	2-3	0.1763/(0.38*((1/0.2584+1/0.3280-(2.9289*$\langle\tau_{D(A)}\rangle_f$ +-0.8539)/(0.3280*0.2584))-1/3.0660))
	3-1	0.1763/(0.38*((1/1.7191+1/0.2580-(2.2776*$\langle\tau_{D(A)}\rangle_f$ +-2.1956)/(0.2580*1.7191))-1/3.0660))
**D91C/A230C-DA**	1-2	0.1691/(0.41*((1/1.9750+1/0.7697-(1.4054*$\langle\tau_{D(A)}\rangle_f$ +-0.7674)/(0.7697*1.9750))-1/3.3669))
	2-3	0.1691/(0.41*((1/2.8398+1/1.9750-(0.9357*$\langle\tau_{D(A)}\rangle_f$

		+0.2506)/(1.9750*2.8398))-1/3.3669))
	3-1	0.1691/(0.41*((1/0.7697+1/2.8400-(1.3289*$\langle\tau_{D(A)}\rangle_f$ +-0.9082)/(2.8400*0.7697))-1/3.3669))
**M149C/A230C-DA**	1-2	0.1767/(0.41*((1/2.4741+1/1.8263-(1.1220*$\langle\tau_{D(A)}\rangle_f$ +-0.2544)/(1.8263*2.4741))-1/3.6193))
	2-3	0.1767/(0.41*((1/0.3820+1/2.4740-(1.9521*$\langle\tau_{D(A)}\rangle_f$ +-2.3469)/(2.4740*0.3820))-1/3.6193))
	3-1	0.1767/(0.41*((1/1.8263+1/0.3820-(2.0101*$\langle\tau_{D(A)}\rangle_f$ +-1.8497)/(0.3820*1.8263))-1/3.6193))
**S142C/K202C-DA**	1-2	0.1877/(0.42*((1/2.4587+1/3.4466-(1.1154*$\langle\tau_{D(A)}\rangle_f$ +-0.3782)/(3.4466*2.4587))-1/4.1254))
	2-3	0.1877/(0.42*((1/0.3370+1/2.4590-(2.3042*$\langle\tau_{D(A)}\rangle_f$ +-3.2171)/(2.4590*0.3370))-1/4.1254))
	3-1	0.1877/(0.42*((1/3.4466+1/0.3370-(2.2527*$\langle\tau_{D(A)}\rangle_f$ +-4.3154)/(0.3370*3.4466))-1/4.1254))

Supplementary Methods

Ensemble Time Correlated Single Photon Counting (eTCSPC) analysis

Time resolved fluorescence decays ($F(t)$) were described using a multi-exponential model as follows

$$F(t) = \sum_i x^{(i)} \exp\left(-\frac{t}{\tau^{(i)}}\right), \quad (1)$$

where $x^{(i)}$ is the i-th population fraction and $\tau^{(i)}$ is the fluorescence lifetime of that population. The average lifetimes for each species and the overall average fluorescence lifetimes are

$$\langle\tau\rangle_x = \sum_i x^{(i)} \cdot \tau^{(i)} \text{, and } \langle\tau\rangle_f = \frac{\sum_i x^{(i)} \cdot \left(\tau^{(i)}\right)^2}{\langle\tau\rangle_x}, \quad (2)$$

respectively.

To model a superposition of multiple Gaussian-distributed FRET states, with population fractions "*j*" corresponding to $x_{DA}^{(j)}$, one needs to generate the interdye distance distribution $p(R_{DA})$ as follows

$$p(R_{DA}) = \sum_{j=1,2,3} x_{DA}^{(j)} \frac{1}{\sqrt{2\pi} \cdot \sigma_{DA}} \exp\left(-\frac{\left(R_{DA} - \langle R_{DA}^{(j)}\rangle\right)^2}{2\,\sigma_{DA}^2}\right), \quad (3)$$

where σ_{DA} is the width of the distribution, which comes from the Accessible Volume of the fluorescent dyes. $\langle R_{DA}\rangle$is the mean interdye distance. The final model included a no FRET state, whose fluorescence decay is the same as that for the donor-only $\left(D_{(0)}\right)$ control.

$$F(t) = \sum_{i=1,2} x_{D(0)}^{i} \exp\left(-\frac{t}{\tau_{D(0)}^{(i)}}\right) \cdot \sum_{j=1,2,3} x_{DA}^{(j)} \int p\left(R_{DA}^{(j)}\right) \exp\left(-t \cdot k_{RET}^{(j)}\right) dR_{DA}, \quad (4)$$

where $k_{RET}^{(j)}$ is the rate of energy transfer of the j-th FRET state and is a function of the interdye distance

$$k_{RET} = k_F \langle\kappa^2\rangle \left(\frac{R_0}{R_{DA}}\right)^6, \quad (5)$$

where $k_F$is the radiative rate constant, R_0 is the Förster radius, $\langle\kappa^2\rangle$ is the interdye orientation factor. The isotropic assumption ($\langle\kappa^2\rangle = \frac{2}{3}$) was verified by time resolved anisotropy.

To obtain the time-resolved anisotropy ($r(t)$), fluorescence intensity decays in both polarizations ($F_{\parallel}$ and $F_{\perp}$ with respect to the excitation source) were collected to determine the proper detection-efficiency factor as

$$F_{\parallel}(t) = \frac{1}{3}F(t)[1 + 2r(t)], \qquad F_{\perp}(t) = \frac{1}{3}F(t)[1 - r(t)] \quad (6)$$

Time-resolved anisotropy and fluorescence intensity decays for both polarizations were jointly fit using a multi-exponential decay function

$$r(t) = \frac{F_{\parallel}(t) - F_{\perp}(t)}{F_{\parallel}(t) + 2 \cdot G \cdot F_{\perp}(t)} \quad (7)$$

$$r(t) = \Sigma_i b_i \exp\left(-\frac{t}{\rho_i}\right)$$

where b_i is the fractional anisotropy, ρ is the rotational correlation time, and *G* is a detection correction parameter (i.e. *G*-factor).

Accessible Volume (AV) simulations to estimate measured distance

To account for dye linker mobility, we calculated AVs for donor and acceptor dyes attached to each labeling site. The AV approximates the dye as a hard sphere connected to the protein via a linker modeled as a flexible cylinder with the dimensions based on the chemical structure [1-4]. For Alexa 488, the five-carbon linker length was set to 20 Å, the width of the linker is 4.5 Å and three dye radii 5.0 Å, 4.0 Å, and 1.5 Å. Similarly, for

Alexa 647 the dimensions used were: length = 22 Å, width = 4.5 Å and the three dye radii 11.0 Å, 3.0 Å, and 1.5 Å.

For each FRET pair, we calculated the distance between dye mean positions (⟨R_{DA}⟩) within the AV.

$$\langle R_{DA}\rangle = \left|\langle R_D^{(i)} - R_A^{(j)}\rangle\right| = \frac{1}{nm}\sum_{i=1}^{n}\sum_{j=1}^{m}\left|R_D^{(i)} - R_A^{(j)}\right|, \quad (8)$$

where $\overrightarrow{R_D^{(i)}}$ and $\overrightarrow{R_A^{(j)}}$ are all the possible positions (*n*,*m*) that the donor and acceptor fluorophores adopt.

Analysis of Multiparameter Fluorescence Detection (MFD) for single-molecule FRET (smFRET) experiments

Single molecule events were identified as events with intensity 2σ above the mean background signal. Cut-off times varied from sample to sample with a minimum of 60 photons collected for each burst [5,6]. Each burst was then processed and fit using a maximum-likelihood algorithm as previously described [7]. The single molecule selection rules are: the difference in single molecule duration on green channels given donor excitation (T_{GX}) and duration on red channels given direct acceptor excitation (T_{RR}) was -1.5 ms < T_{GX}-T_{RR} < 1.5 ms; and satisfy the FRET Stoichiometry (S_{PIE}) parameter of 0.13 < S_{PIE} < 0.6 , which selects for bursts which both fluorophores present. The PIE stoichiometry is given by:

$$S_{PIE} = \frac{F_{R|D} - \beta F_{R|A} - \alpha F_{G|D} + \gamma F_{G|D}}{F_{R|D} - \beta F_{R|A} - \alpha F_{G|D} + \gamma F_{G|D} + F_{R|A}}, \quad (9)$$

where $F_{G|D}$, $F_{R|D}$, and $F_{R|A}$ are background-corrected fluorescence intensities measured in the donor channel after donor excitation ($G|D$), in the acceptor channel after donor excitation ($R|D$) and direct acceptor excitation ($R|A$), respectively, α is the correction factor for donor crosstalk into the acceptor channel, β is the correction factor for direct excitation of the acceptor by the donor excitation source, and $\gamma = \frac{\Phi_{FA}}{\Phi_{FD(0)}} \cdot \frac{g_R}{g_G}$ is a function of the quantum yield of the donor $\Phi_{FD(0)}$, the quantum yield of the acceptor Φ_{FA} and detection efficiencies *g*. Fluorescent bursts were plotted in 2D histograms (Origin 8.6, OriginLab Co).

Calculation of the Static and Dynamic FRET Lines

The relationship between FRET intensity (F_D/F_A) and the FRET lifetime ⟨$\tau_{D(A)}$⟩ is different for static molecules and those undergoing dynamic averaging. To facilitate interpretation of the MFD experiments, we include guidelines that relate FRET intensity (F_D/F_A) to the FRET lifetime ⟨$\tau_{D(A)}$⟩ for molecules that are static and those that are dynamic. The parametric line that relates the FRET indicators, $E_{static,L}$ (i.e. FRET Efficiency) or $(F_D/F_A)_{static,L}$ and $\langle\tau_{D(A)}\rangle_f$, in the absence of dynamics is defined as

$$E_{static,L} = 1 - \frac{\sum_{i=0} A^{(i)} \cdot \langle\tau_{D(A)}\rangle_f^i}{\tau_{D(0)}}$$

$$\left(\frac{F_D}{F_A}\right)_{static,L} = \left(\gamma' \cdot \left(\frac{\tau_{D(0)}}{\sum_{i=0} A^{(i)} \cdot \langle\tau_{D(A)}\rangle_f^i} - 1\right)\right)^{-1}, \quad (10)$$

where A_i are the coefficients of an empirical polynomial function that takes into account the intrinsic linker dynamics (*L*) of the dyes, and $\gamma' = \Phi_{FA}/\Phi_{FD(0)}$, which is the ratio of the quantum yields of acceptor to donor. Hereafter, we used the simplified notation of $F_{G|D} = F_D$ and $F_{R|D} = F_A$.

The parametric line that relates the FRET indicators in the presence of dynamics is defined as

$$E_{dyn,L} = 1 - \frac{\langle\tau_{D(A)}^{(1)}\rangle_f \cdot \langle\tau_{D(A)}^{(2)}\rangle_f}{\tau_{D(0)}\left(\langle\tau_{D(A)}^{(1)}\rangle_f + \langle\tau_{D(A)}^{(2)}\rangle_f - \sum_{i=0}^{3} C_{i,L}\left(\langle\tau_{D(A)}\rangle_f\right)\right)^i} \quad (11)$$

$$\left(\frac{F_D}{F_A}\right)_{dyn,L} = \frac{1}{\gamma' \cdot \tau_{D(0)}} \cdot \frac{\langle\tau_{D(A)}^{(1)}\rangle_f \cdot \langle\tau_{D(A)}^{(2)}\rangle_f}{\left(\langle\tau_{D(A)}^{(1)}\rangle_f + \langle\tau_{D(A)}^{(2)}\rangle_f - \sum_{i=0}^{3} C_{i,L}\left(\langle\tau_{D(A)}\rangle_f\right)^i\right) - \frac{\langle\tau_{D(A)}^{(1)}\rangle_f \cdot \langle\tau_{D(A)}^{(2)}\rangle_f}{\tau_{D(0)}}},$$

where C_i is the coefficient of an empirical polynomial function that takes into account the intrinsic linker dynamics (*L*) of the dyes. The superscripts (1) and (2) indicate the two limiting states that are in exchange, which will be connected by the dynamic FRET line.

Determination of Quantum Yields

We assume that only dynamic quenching takes place such that $\Phi_{FD(0)}$ and Φ_{FA} are proportional to the species-averaged fluorescence lifetime $\langle\tau_{D(A)}\rangle_x$ of donor and acceptor, respectively. As reference samples we used Alexa488-labeled DNA $\langle\tau_{D(0)}\rangle_x$ = 4.0 ns, $\Phi_{FD(0)}$ = 0.8 and for the acceptor we used Cy5-labeled DNA with $\langle\tau_A\rangle_x$ = 1.17 ns and Φ_{FA} = 0.32 [8]. The obtained donor and acceptor quantum yields are presented in Supplementary Table 1B, 1C. For Alexa488 and Alexa647, we considered a reduced Förster distance of 52 Å where we assumed isotropic reorientation of the dyes using κ^2 = 2/3 due to the long linkers.

Estimation of $\langle\kappa^2\rangle$ and κ^2-distributions along with the associated uncertainty

Experimentally, we probe if the assumption of κ^2 = 2/3 is justifiable or not. Considering that fluorophores follow the "wobble-in- cone" model [9], it is possible to calculate a distribution of all possible values of κ^2. For that, we need the residual anisotropies obtained by fitting the time resolved anisotropy ($r_\infty = b_3$, *Eq. 7*) for the various sources (Donly - donor, A - acceptor and A(D) – FRET-sensitized emission of acceptor). Then, all κ^2 values can be described as follows.

$$\begin{aligned}\kappa^2 = \frac{2}{3} + \frac{2}{3}S_D^{(2)}S^{(2)}(\beta_1) + \frac{2}{3}S_A^{(2)}S^{(2)}(\beta_2) \\ + \frac{2}{3}S_D^{(2)}S_A^{(2)}\big(S^{(2)}(\delta) + 6S^{(2)}(\beta_1)S^{(2)}(\beta_2) + 1 + 2S^{(2)}(\beta_1) + 2S^{(2)}(\beta_1) + 2S^{(2)}(\beta_2) \\ - 9cos\beta_1 cos\beta_2 \cos\delta\big)\end{aligned} \quad (12)$$

where β_1 and β_2 are the angles between the symmetry axes of each dye rotation, and δ is the angle between them. The necessary second-rank order parameters $S^{(2)}$ are defined by:

$$\begin{aligned} S^{(2)}(\delta) &= \frac{1}{2}(3\cos^2\delta - 1) = \frac{r_{\infty,A(D)}}{r_0 S_D^{(2)} S_A^{(2)}} \\ S^{(2)}(\beta_1) &= \frac{1}{2}(3\cos^2\beta_1 - 1) \text{ and} \\ S^{(2)}(\beta_2) &= \frac{1}{2}(3\cos^2\beta_2 - 1)\end{aligned} \quad (13)$$

where r_0 is the fundamental anisotropy of the dyes, which were 0.38 and 0.39 for the donor and acceptor fluorophores, respectively [4]. The dye motions are characterized by the second-rank order parameters $S_D^{(2)}$ and $S_A^{(2)}$ as

$$\begin{aligned} \text{Donor:} \quad & \frac{1}{2}\cos^2\theta_{\text{disk}} = \sqrt{\frac{r_{\infty,Donly}}{r_0}} = -S_D^{(2)} \\ \text{Acceptor:} \quad & \frac{1}{2}\cos^2\theta_{\text{cone}}(1 + \cos\theta_{cone}) = \sqrt{\frac{r_{\infty,A}}{r_0}} = S_A^{(2)}, \end{aligned} \quad (14)$$

From all possible orientations and combinations, a κ^2- distribution and its corresponding arithmetic mean ($\langle\kappa^2\rangle$) can be determined and compared to the assumed κ^2=2/3 (Fig. 3).

Error propagation

To estimate the statistical uncertainty in the experimental distances, we used an error propagation rule, which considered the uncertainty associated with the minimization of χ^2,($\Delta\chi^2$), along with the uncertainty associated with the unknown orientation of the dyes, ($\Delta\kappa^2$). For $\Delta\chi^2$, we first calculate the maximum allowed $\chi^2_{r,max}$ for a given confidence-level (*P;* e.g. for 2σ or *P* = 0.95) as

$$\chi^2_{r,max}(P) = \chi^2_{r,min} \cdot \left[1 + \frac{n}{v} \cdot cdf^{-1}\big(F(n, v, P)\big)\right], \tag{15}$$

given the minimum $\chi^2_{r,min}$ obtained by the fit and identified the distances R_{DA} at these particular values of χ^2. We then obtain $\Delta\kappa^2$ by determining the arithmetic mean of the calculated κ^2 distribution. Then, the overall uncertainty is determined as

$$\varepsilon(\kappa^2, k_{FRET}) = \Delta R^2_{DA}(\kappa^2) + \Delta R^2_{DA}(k_{FRET}), \tag{16}$$

which is presented in Supplementary Table 1.

Global Analysis and goodness of fit.

To test the goodness of the fit for the fluorescence decays, we rely on i) the visual inspection of residuals ii) the autocorrelation of the residuals; and ii) the F-test ([10,11]. The F-test compares the ratio of two independent χ^2variables, each with their own numbers of degrees of freedom with a similar equation as the one shown in Eq. 15.

The global analysis improved χ^2 from 3- to 2-FRET distributed states by 0.15. This change, given the changes in the number of degrees of freedom, corresponds to a confidence of 99.99% that the 3-FRET distributed states better describes the experimental observations. Particularly because the number of degrees of freedom is significantly reduced from an independent fit to a global analysis. Additionally, the global analysis allows for an unbiased assignment of distances to particular configuration states, based on the population fractions.

References

1. Sindbert, S. et al. *Journal of the American Chemical Society* **133** (8), 2463 (2011).
2. Cai, Q. et al. *Biophys J* **93** (6), 2110 (2007).
3. Muschielok, A. et al. *Nat Methods* **5** (11), 965 (2008).
4. Kalinin, S. P., T.; Sindbert, S.; Rothwell, P. J.; Berger, S.; Restle, T.; Goody, R. S.; Gohlke, H.; Seidel, C. A. M. *Nature Methods* (2012).
5. Kühnemuth, R. and Seidel, C. A. M. *Single Molecules* **2** (4), 251 (2001).
6. Schaffer, J. et al. *Journal of Physical Chemistry A* **103** (3), 331 (1999).
7. Maus, M. et al. *Anal Chem* **73** (9), 2078 (2001).
8. Woźniak, A. K. et al. *Proc.Natl.Acad.Sci.USA.* **105**, 18337 (2008).
9. Dale, R. E., Eisinger, J., and Blumberg, W. E. *Biophys J* **26** (2), 161 (1979).
10. Box George, E. P. *Annals of the New York Academy of Sciences* **86** (3), 792 (1960).
11. Lakowicz, J. R., (Springer US, 2006).
12. Sisamakis, E. et al. *Methods in enzymology* **475**, 455 (2010).
